# An Update of Mobile Colistin Resistance in Non-Fermentative Gram-Negative Bacilli

**DOI:** 10.3389/fcimb.2022.882236

**Published:** 2022-06-17

**Authors:** Piyatip Khuntayaporn, Krit Thirapanmethee, Mullika Traidej Chomnawang

**Affiliations:** ^1^ Department of Microbiology, Faculty of Pharmacy, Mahidol University, Bangkok, Thailand; ^2^ Antimicrobial Resistance Interdisciplinary Group (AmRIG), Faculty of Pharmacy, Mahidol University, Bangkok, Thailand

**Keywords:** colistin resistance, *mcr* genes, *Acinetobacter*, *Pseudomonas*, polymyxin

## Abstract

Colistin, the last resort for multidrug and extensively drug-resistant bacterial infection treatment, was reintroduced after being avoided in clinical settings from the 1970s to the 1990s because of its high toxicity. Colistin is considered a crucial treatment option for *Acinetobacter baumannii* and *Pseudomonas aeruginosa*, which are listed as critical priority pathogens for new antibiotics by the World Health Organization. The resistance mechanisms of colistin are considered to be chromosomally encoded, and no horizontal transfer has been reported. Nevertheless, in November 2015, a transmissible resistance mechanism of colistin, called mobile colistin resistance (MCR), was discovered. Up to ten families with MCR and more than 100 variants of Gram-negative bacteria have been reported worldwide. Even though few have been reported from *Acinetobacter* spp. and *Pseudomonas* spp., it is important to closely monitor the epidemiology of *mcr* genes in these pathogens. Therefore, this review focuses on the most recent update on colistin resistance and the epidemiology of *mcr* genes among non-fermentative Gram-negative bacilli, especially *Acinetobacter* spp. and *P. aeruginosa*.

## 1 Introduction

Presently, beta-lactams, cephalosporins, carbapenems, fluoroquinolones, aminoglycosides, and macrolides are frequently used to treat bacterial infections. However, the emergence of drug-resistant microorganisms, particularly Gram-negative pathogens, has become a public health threat. In 2017, the World Health Organization classified carbapenem-resistant (CR) *Acinetobacter baumannii* and *Pseudomonas aeruginosa* as priority pathogens in critical need of alternative treatment options (World Health Organization, 2017). *P. aeruginosa*, *A. baumannii*, *Stenotrophomonas maltophilia*, and *Burkholderia cepacia* complex are non-fermentative Gram-negative bacteria that cause significant problems in healthcare settings. Because these bacteria are highly adaptable and have various intrinsic and acquired resistance mechanisms, they are typically resistant to major classes of antimicrobial agents, leaving only a few therapeutic options (Enoch et al., 2007). Among these, *P. aeruginosa* and *A. baumannii* are the most common causes of nosocomial infections (Mancuso et al., 2021). *P. aeruginosa* is the most common pathogen in the *Pseudomonas* genus. This bacterium is an opportunistic pathogen that causes skin, wound, and lung infections. Respiratory infections caused by *P. aeruginosa* are often associated with defective respiratory systems or ventilation, such as in cystic fibrosis (Bassetti et al., 2018). In contrast, *A. baumannii* is one of the most common causes of nosocomial infections, such as bloodstream infections and pneumonia (Garnacho-Montero and Timsit, 2019). This organism is part of what is known as the *Acinetobacter calcoaceticus–baumannii* complex, which also includes *Acinetobacter pittii*, *Acinetobacter nosocomialis*, and *Acinetobacter calcoaceticus* (Ramirez et al., 2020).

With the limitations of new drug development, many outdated antibiotics have been reintroduced into the clinical setting despite their high toxicity, including the polymyxin drug group (Hermsen et al., 2003). As there are no other options available, this drug group has become crucial to combat antibiotic resistance (Bialvaei and Samadi Kafil, 2015). Modern therapeutic drug monitoring of colistin is prone to have a lower incidence rate of toxicity when compared to the past.

The polymyxin group contains many drugs, but polymyxin E, also known as colistin, is recognized as the main agent (Rhouma et al., 2016b). Colistin is one of the remaining treatment options for life-threatening infections caused by multidrug and extensively drug-resistant *A. baumannii* and *P. aeruginosa* (Bialvaei and Samadi Kafil, 2015). Moreover, colistin resistance mechanisms are quite rare and chromosomally encoded, which makes transfer difficult (Olaitan et al., 2014). Therefore, the resistance rate against colistin in Gram-negative pathogens appears to be lower than that of other antibiotic classes. However, the increasing trend of colistin resistance in Enterobacteriaceae led to the discovery of the transmissible resistance mechanism of colistin in 2015 (Liu et al., 2016). Since then, the resistance rate of last-resort drugs has been closely monitored; the more the antibiotic resistance rate increases, the fewer treatment options are available. Transferable polymyxin resistance has been extensively reported worldwide. To date, at least ten variations in *mcr* genes have been described and are currently ongoing. This problem is critical, especially for pathogens with limited treatment options, such as *A. baumannii* and *P. aeruginosa*. Therefore, this review focuses on colistin drug resistance and its epidemiology among the non-fermentative Gram-negative bacilli, *Acinetobacter* spp., and *P. aeruginosa*.

## 2 The Polymyxins

**Figure 1:**
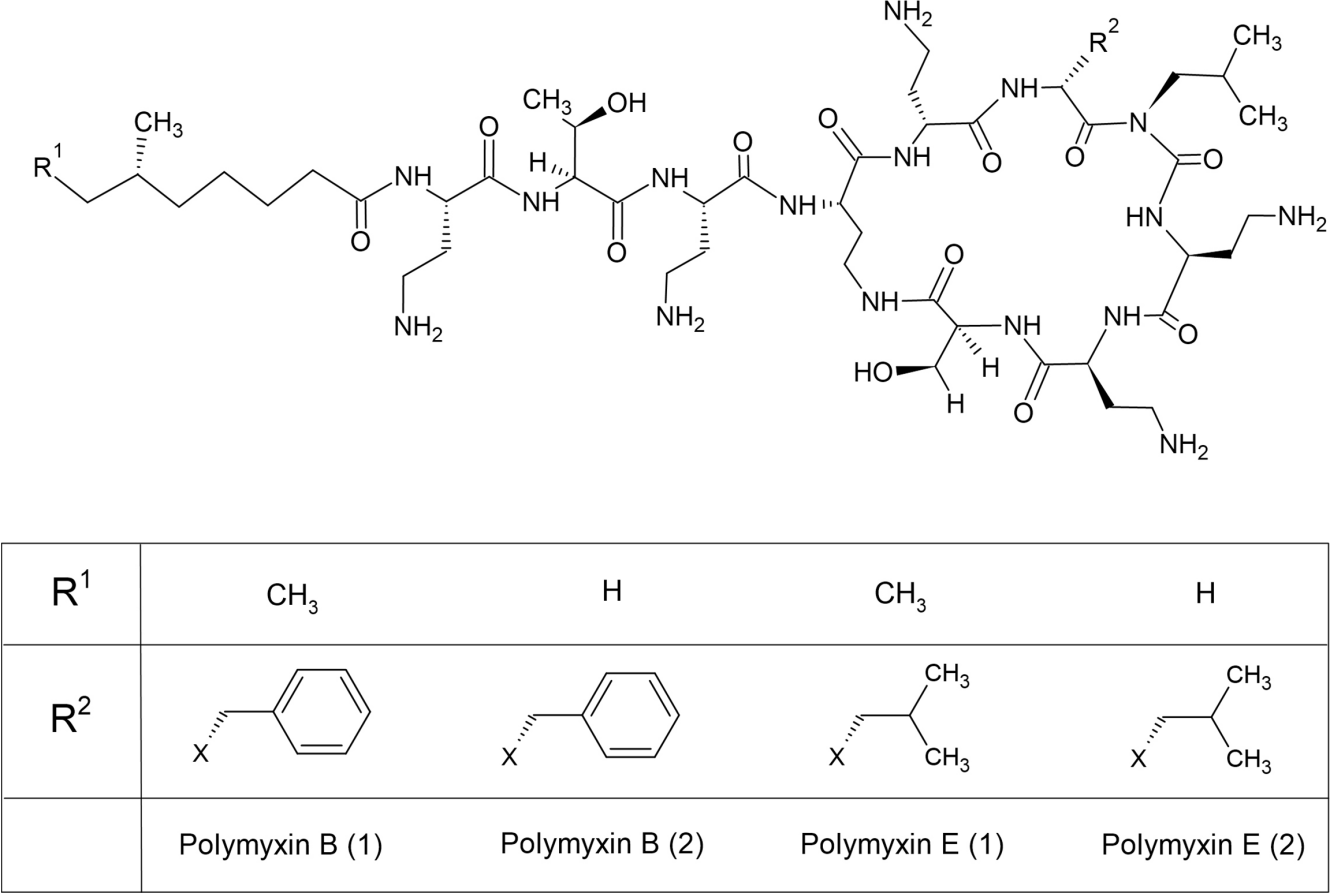
Chemical structures of polymyxin B and E.

Colistin (polymyxin E) belongs to the polymyxin drug group and appears commercially in two forms as inactive prodrugs: colistin methanesulfonate for parenteral use and colistin sulfate for topical use and use in animal production in some countries (Rhouma et al., 2016b). Another type of polymyxin used in clinical practice is polymyxin B, which is administered in its active form (Tsuji et al., 2019). These antibiotics have been described as old-generation antibiotics, but because of the limitations of antibiotic options, colistin was reintroduced as a last resort for multidrug-resistant (MDR) and extensively drug-resistant (XDR) bacterial infection treatment. Polymyxins were discovered in the 1940s from *Bacillus polymyxa*, later known as *Paenibacillus polymyxa*, and were approved by the United States Food and Drug Administration before being used in hospitals in the 1950s (Lim et al., 2010). Polymyxins are polypeptide antibiotic groups that include five different chemical compounds: polymyxins A, B, C, D, and E; however, only polymyxin B and polymyxin E are used in clinical settings (Bialvaei and Samadi Kafil, 2015). Polymyxin B consists of two compounds, polymyxins B1 and B2, whereas colistin contains polymyxins E1 and E2. Colistin differs from polymyxin B in its amino acid composition (**Figure 1**) (Hermsen et al., 2003; Nation and Li, 2009; Bialvaei and Samadi Kafil, 2015). It has a molecular weight of 1,750 Da and consists of a polycationic cyclic heptapeptide attached to a lipophilic fatty acid side chain (Bialvaei and Samadi Kafil, 2015). The structure of colistin is amphipathic, containing both aqueous and non-aqueous soluble parts (Hermsen et al., 2003).

Colistin has been demonstrated to have a concentration-dependent bactericidal effect, but its mechanism of action is unclear (Nation and Li, 2009; Bialvaei and Samadi Kafil, 2015). The proposed mechanism of action is based on the chemical structure of colistin, which destabilizes lipopolysaccharide (LPS), increases membrane permeability, and leads to bacterial cell leakage (Hermsen et al., 2003; Nation and Li, 2009). The antibiotic spectrum of colistin is narrow, but it is active against many important MDR Gram-negative bacteria, including *P. aeruginosa*, *A. baumannii*, *Escherichia coli*, *Klebsiella pneumoniae*, *Enterobacter* spp., and some other bacteria in Enterobacterales (Hermsen et al., 2003; Nation and Li, 2009; Bialvaei and Samadi Kafil, 2015). Colistin is generally not recommended for Gram-positive pathogens because they lack an outer membrane structure.

Its prominent toxicity, including nephrotoxicity and neurotoxicity, is a drawback of colistin use (Nation and Li, 2009). However, toxicity is usually reversible upon discontinuing the medication and is believed to be dose dependent (Li et al., 2006). In the early 2000s, when a resurgence of colistin use occurred, the lack of information on appropriate colistin dosage was the main problem. The International Consensus Guidelines for the Optimal Use of Polymyxins were published in 2020, making colistin safer for use. The recommended PK/PD therapeutic target for efficacy maximization of colistin is a target plasma colistin C_ss,avg_ of 2 mg/L, which can provide an area under the plasma concentration–time curve across 24 h at a steady state (AUC_ss,24 h_) of approximately 50 mg h/L (Tsuji et al., 2019). This concentration is considered the maximum tolerable exposure. Higher concentrations can increase the nephrotoxicity incidence and severity (Tsuji et al., 2019). Patients with renal impairment should have their colistin dosage adjusted based on creatinine clearance (Tsuji et al., 2019).

## 3 The Laboratory Detection of Colistin Resistance

The phenotypic detection of colistin resistance is usually based on antimicrobial susceptibility testing. Clinical and Laboratory Standards Institute (CLSI) guidelines recommend that the broth dilution method be used for colistin because the disc diffusion method is unreliable (Falagas et al., 2010). As a result, the CLSI-EUCAST Working Group recommended a reference method for polymyxin susceptibility testing using broth microdilution without additives (Dafopoulou et al., 2019). The microbe is considered colistin or polymyxin B resistant when the minimum inhibitory concentration (MIC) is equal to or greater than 4 mg/ml in tested organisms, including in Enterobacterales, *P. aeruginosa*, and *Acinetobacter* spp. (Clinical and Laboratory Standards Institute, 2022). Colistin resistance in Enterobacterales and *Acinetobacter* spp. is when the MIC is greater than 2 mg/ml, but resistance in *P. aeruginosa* is when the MIC is greater than 4 mg/ml, according to the EUCAST breakpoints table (The European Committee on Antimicrobial Susceptibility Testing, 2022). Additionally, sulfate salts of polymyxins must be used instead of colistin methanesulfonate because of their slow breakdown from the inactive prodrug form (Tsuji et al., 2019). Agar dilution is another antimicrobial susceptibility method based on dilution techniques. However, the reliability of the MICs obtained using this method remains inconclusive. Therefore, the CLSI-EUCAST Working Group advised to avoid using the agar dilution method until more data are available (Dafopoulou et al., 2019). Notably, the phenotypic detection method cannot distinguish between colistin resistance mechanisms. To identify the resistance mechanisms, analyses at the genotypic level should be applied subsequent to the antimicrobial susceptibility test. Genotypic detection is based on polymerase chain reaction (PCR) and whole-genome sequencing (WGS) methods. WGS seems to be the most effective strategy for collecting these data because it can identify all targeted antimicrobial resistance genes, including acquired colistin resistance genes (World Health Organization, 2020). The PCR detection method has limitations owing to its selective amplification of only the known sequence. If suspected organisms carry novel *mcr* genes or mutations, the PCR method alone may not detect that information (World Health Organization, 2021).

Since the presence of *mcr* genes on transmissible plasmids was reported, the colistin resistance rate has increased significantly, especially in Asia, Africa, and Europe. Therefore, rapid screening methods for *mcr*-harboring microorganisms are necessary. The only recommended phenotypic detection method is broth dilution, which is laborious compared to the disc diffusion or gradient diffusion methods. Although genetic-based detection methods are the gold standard, they require sophisticated instruments and experienced users. It is also difficult to detect all the responsible colistin resistance genes, especially the acquired genes. Therefore, a more practical method for routine laboratory screening is required (World Health Organization, 2021). Phenotypic detection methods that are still under development have been proposed, such as agar-based screening media (CHROMID^®^ Colistin R agar, Superpolymyxin™, CHROMagar™ COL-APSE), the Rapid Polymyxin NP test, Colispot, and disc prediffusion (Boyen et al., 2010; Nordmann et al., 2016a; Nordmann et al., 2016b; Abdul Momin et al., 2017; Jouy et al., 2017; Garcia-Fernandez et al., 2019).

## 4 Colistin Resistance Surveillance

Public health awareness of the increasing prevalence of antimicrobial-resistant microorganisms has led to the implementation of antimicrobial stewardship programs worldwide. One strategy is to monitor the resistance of bacteria to slow the spread of resistant microorganisms. Therefore, many surveillance programs have been initiated to monitor antimicrobial resistance in all countries, including the Global Antimicrobial Resistance and Use Surveillance System (GLASS), Central Asian and European Surveillance of Antimicrobial Resistance (CAESAR), Latin American and Caribbean Network for Antimicrobial Resistance Surveillance (ReLAVRA), and the European Antimicrobial Resistance Surveillance Network (EARS-Net). A report of colistin resistance in bloodstream infections from the SENTRY program from 2009 to 2016 showed a resistance rate of less than 1% in *P. aeruginosa*, 3.1% in *A. baumannii*, and more than 10% in Enterobacteriaceae (Diekema et al., 2019). In Canada, the CANWARD surveillance study showed that between 2007 and 2016, *Enterobacter cloacae*, *P. aeruginosa*, and *A. baumannii* were the top three microorganisms with the highest colistin resistance rates of approximately 18.1%, 5.0%, and 2.5%, respectively (Zhanel et al., 2019). Similar results were obtained by Bialvaei and Kafil, who also detected a high resistance rate of colistin among Enterobacteriaceae, especially from *Enterobacter* spp. and *K. pneumoniae*, in the Asia-Pacific and Latin American regions (Bialvaei and Samadi Kafil, 2015). The abrupt increase in colistin resistance in Asian countries has led to the discovery of mobile colistin resistance (MCR). In Thailand, the National Antimicrobial Resistance Surveillance Center, Thailand (NARST) also monitors colistin resistance in clinically important microorganisms. Fortunately, the resistance rates to colistin in *E. coli*, *K. pneumoniae*, *P. aeruginosa*, and *A. baumannii* in Thailand in 2019 were less than 5% (National Antimicrobial Resistant Surveillance Center, 2020).

## 5 The Importance of the Polymyxins in Non-Fermentative Bacteria Treatment

Polymyxins and carbapenems are considered last-resort antibiotics for the treatment of Gram-negative bacteria; however, owing to their misuse, the problem of antibiotic resistance is worsening, particularly in low- to middle-income countries (de Carvalho et al., 2022). A multicenter surveillance study in Taiwan found that the incidence of MDR, XDR, and CR *P. aeruginosa* infections in hospitalized patients increased from 25.1% to 27.5%, 7.7 to 8.4%, and 19.7% to 27.5%, respectively, between 2016 and 2018 (Jean et al., 2022). In the past decade, hospital-associated *P. aeruginosa* has showed high MDR/CR numbers in Europe, with prevalence rates of more than 30% (Micek et al., 2015). In 2020, more than half of the countries in Europe showed carbapenem resistance of more than 25% among invasive isolates (European Centre for Disease Prevention and Control, 2022). A meta-analysis found that colistin is the most effective antibiotic for the treatment of *Pseudomonas* spp. Throughout the study period, colistin was the only antibiotic with a resistance rate of less than 10% (Bonyadi et al., 2022).

In China, *Acinetobacter* spp. showed a high level of resistance to all carbapenems caused by plasmids carrying various carbapenemase genes (Jean et al., 2022). In a 2022 report from Europe, healthcare-associated isolates of CR-*Acinetobacter* spp. were >50% in at least 20 countries, especially in southern and eastern Europe (European Centre for Disease Prevention and Control, 2022). Data from many surveillance studies have indicated that carbapenem resistance has been increasing over the last decade, suggesting that carbapenems may not be a suitable standard treatment for MDR, XDR, and CR non-fermentative Gram-negative bacteria. Therefore, polymyxin-based therapy has become the recommended treatment option for CR *A. baumannii* (CRAB) and XDR *P. aeruginosa* infections (de Carvalho et al., 2022). In clinical practice, colistin or polymyxin has always been used in combination therapy with at least one additional antibiotic from a different class against CR microorganisms or in patients with risk factors (Bassetti et al., 2018; Doi 2019).

The “Guidelines Recommendations for Evidenced-based Antimicrobial use in Taiwan” (GREAT) working group has launched recommendations and guidelines for the treatment of infections caused by MDR organisms (Sy et al., 2022). In bloodstream infections caused by CRAB, the recommended treatment is colistin 5 mg/kg IV loading dose, followed by IV every 12 h of 2.5 mg × (1.5 × creatine clearance + 30) and/or imipenem/cilastatin 500 mg IV every 6 h or meropenem 2 g IV every 8 h. In pneumonia caused by CRAB, the recommended treatment is colistin 5 mg/kg IV loading dose, then IV every 12 h of 2.5 mg × (1.5 × creatine clearance + 30) and/or imipenem/cilastatin 500 mg IV every 6 h or meropenem 2 g IV every 8 h and adjunctive colistin inhalation 1.25–15 MIU/day in 2–3 divided doses. For any clinical symptoms caused by difficult-to-treat *P. aeruginosa*, one of the recommended regimens is colistin 5 mg/kg IV loading dose, followed by IV every 12 h at 2.5 mg × (1.5 × creatine clearance + 30) or combination therapy for 5–14 days. Colistin plays a crucial role in MDR microorganism treatment. Therefore, if colistin resistance mechanisms can be transmitted more easily like MCR, it would significantly impact non-fermentative Gram-negative bacterial treatment.

## 6 Chromosomal Resistance of Colistin

Before the 2000s, reports of resistance to colistin were quite rare, which might have been caused by its low usage over the last 30 years (Nation and Li, 2009). The main mechanism of polymyxin resistance in Gram-negative bacteria is the modification of lipid A, which reduces electrostatic interactions with polymyxins (Cai et al., 2012). Some Gram-negative bacteria, such as *Proteus* spp. and *Burkholderia* spp., demonstrated resistance to polymyxins naturally by modifying LPS with 4-amino-4-deoxy-L-arabinose (L-Ara4N) (Olaitan et al., 2014). Chromosomal encoding enzymes (EptA, EptB, and EptC) have been identified in some Gram-negative bacteria, such as Salmonella. EptA, also known as PmrC, is a complex operon. These enzymes, encoded by phosphoethanolamine (pEtN) transferases, can add pEtN to LPS (Zhang et al., 2019; Hamel et al., 2021).

The acquired resistance mechanisms of chromosomally encoded polymyxins are mainly caused by modification of the LPS charge (Olaitan et al., 2014). These resistance mechanisms have been reported in many Gram-negative microorganisms, such as *Salmonella enterica*, *K. pneumoniae*, *A. baumannii*, *P. aeruginosa*, and *E. coli*. They are involved in the two-component system genes *phoP*/*phoQ* and *pmrA*/*pmrB* (Needham and Trent, 2013; Olaitan et al., 2014). PhoQ and PmrB proteins possess tyrosine kinase activity, which phosphorylates the regulator protein (PhoP or PmrA), activates the *pmrHFIJKLM* operon, and finally modifies the surface of bacteria by adding L-Ara4N or pEtN to lipid A (Ayoub Moubareck, 2020). PhoP/PhoQ is also regulated by the ColR/ColS and CprR/CprS systems. Mutations in these regulatory systems can lead to overexpression of PhoP/PhoQ in *P. aeruginosa* (Gutu et al., 2013). ParR/ParS is also involved in colistin resistance in *P. aeruginosa*, with upregulation of the LPS modification operon at sub-inhibitory concentrations of polymyxins (Fernandez et al., 2010). The two-component systems found in *P. aeruginosa* are PhoP/PhoQ and PmrA/PmrB, but only PmrA/PmrB has been reported in *A. baumannii* (McPhee et al., 2003; Adams et al., 2009; Beceiro et al., 2011). In addition, in *A. baumannii*, the insertion of ISAba11 into the biosynthesis genes *lpxA*, *lpxC*, and *lpxD* leads to the complete loss of LPS and colistin resistance (Moffatt et al., 2010; Moffatt et al., 2011).

Additional resistance mechanisms, such as overexpression of efflux pumps, outer membrane remodeling, and lack of LPS formation, have also been reported to be involved in colistin resistance (Olaitan et al., 2014; Ayoub Moubareck, 2020). However, these resistance mechanisms appear to be located on the chromosome. Therefore, the transmission of these mechanisms is difficult, and the horizontal gene transfer of these mechanisms has never been reported (Liu et al., 2016).

## 7 Transmissible Resistance of Colistin

Although the use of colistin in human clinical settings was reduced in the 1970s and was reintroduced in the late 1990s, colistin is commonly consumed in animal farming to prevent *E. coli* and *Salmonella* spp. infections (Kempf et al., 2013; Rhouma et al., 2016a). Prior to the discovery of MCR-1, surveillance of antimicrobial resistance revealed a significant increase in colistin resistance. In 2015, the first mobilized colistin resistance gene, *mcr*-1, was discovered in *E. coli* in a Chinese pig farm using a routine antimicrobial resistance surveillance program (Liu et al., 2016). MCR-1 encodes pEtN-lipid A transferase, which can modify the lipid A portion of LPS by the addition of pEtN. MCR-1 also demonstrates transmission and maintenance properties in *K. pneumoniae* and *P. aeruginosa* (Liu et al., 2016). Moreover, the microorganisms that harbored MCR-1 showed an increase in the MIC values of colistin. Furthermore, researchers have identified the *mcr-1* gene in clinical isolates from inpatients in the same area of a pig farm (Liu et al., 2016). Therefore, awareness of this gene**’**s transferable properties is of great concern because colistin is currently considered one of the last-resort treatments for XDR microorganisms.

### 7.1 The Variation of MCR

**Figure 2:**
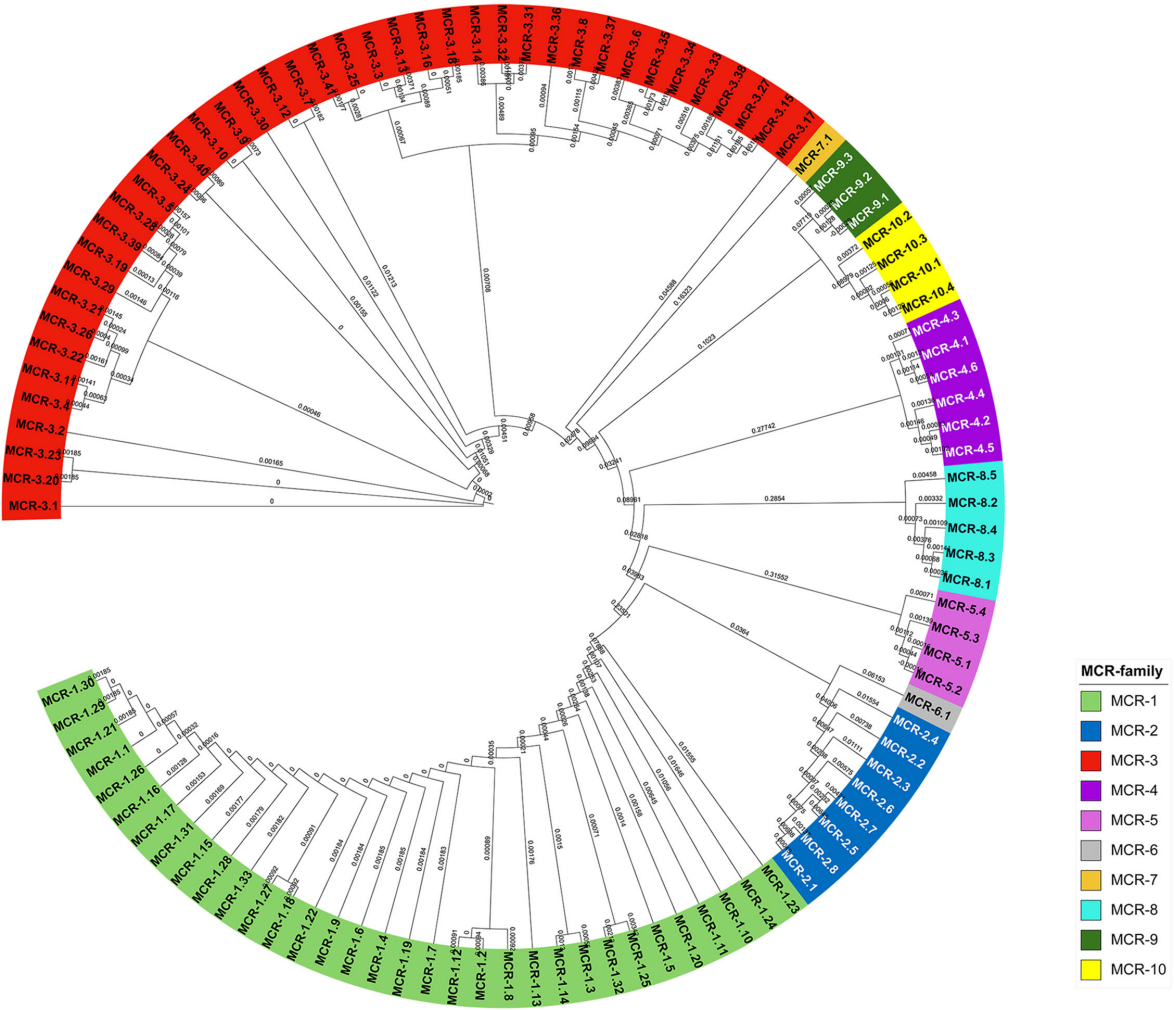
The phylogenetic tree of MCR-gene variants using the Neighbor-Joining method alignment. Multiple sequence alignment was calculated by the clustal omega (Madeira et al., 2019), and the results were illustrated by the Interactive Tree of Life (Letunic and Bork, 2021).

After the MCR-1 discovery, many surveillance programs discovered MCR variation. The nomenclature of *mcr* genes was proposed in 2018 (Partridge et al., 2018). As of January 2022, ten *mcr*-gene families with more than 100 variants have been reported in GenBank. The highest number of MCR variants was found in MCR-3 followed by MCR-1, with 42 and 32 variants, respectively (Medicine, 2022). Only MCR-6 and MCR-7 showed one variant each. MCR-5, MCR-8, and MCR-10 all had four variants. The rest of the MCR families, i.e., MCR-2, MCR-4, and MCR-9, have 8, 6, and 3 variants, respectively. The phylogenetic tree of the MCR families is shown in **Figure 2**.

### 7.2 The Epidemiology of the *mcr* Gene

The discovery of the *mcr* gene occurred from a surveillance study in China before it spread around the world (Liu et al., 2016). In 2016, many countries across all continents except Australia reported the discovery of MCR-1 (**Table 1**). Most MCR-harboring microorganisms belong to the Enterobacterales order, such as *E. coli*, *Salmonella* spp., and *K. pneumoniae*. Apart from Enterobacterales, colistin was also considered a last-resort antibiotic option for non-fermentative Gram-negative bacteria, *Acinetobacter* spp., and *P. aeruginosa*. *Pseudomonas* spp. and *Acinetobacter* spp. are the most common microorganisms that cause nosocomial infections. They have many intrinsic resistance mechanisms and readily acquire transmissible antibiotic resistance genes, which limit antibiotic treatment options. These organisms belong to ESKAPE (*Enterococcus faecium*, *Staphylococcus aureus*, *K. pneumoniae*, *A. baumannii*, *P. aeruginosa*, and Enterobacteriaceae), a group of bacteria that are considered an emerging threat in this century (Boucher et al., 2009). Transferable colistin resistance mechanisms in these organisms are a serious problem in the healthcare setting. Therefore, monitoring the *mcr* gene in non-fermentative Gram-negative bacteria is necessary to combat multidrug resistance.

**Table 1:** List of countries that reported the *mcr-1* gene in 2016.

Continent	Country	List of organisms	Source of specimens	Reference
Africa	Algeria	*E. coli*	A, C	(Berrazeg et al., 2016; Olaitan et al., 2016)
	Egypt	*E. coli*	A, C	(Elnahriry et al., 2016; Khalifa et al., 2016)
	South Africa	*E. coli*	A, C	(Perreten et al., 2016; Poirel et al., 2016)
	Tunisia	*E. coli*	A	(Grami et al., 2016)
Asia	Bahrain	*E. coli*	C	(Sonnevend et al., 2016)
	Cambodia	*E. coli*	C	(Stoesser et al., 2016)
	China	*E. coli* *K. pneumoniae* *E. aerogenes* *E. cloacae* *Kluyvera ascorbata* *S. entirica*	A, C A,C C C E A	(Li et al., 2016; Liu et al., 2016; Zeng et al., 2016; Zhao and Zong, 2016)
	Japan	*E. coli* *S. enterica*	A A	(Kusumoto et al., 2016; Suzuki et al., 2016)
	Laos	*E. coli* *K. pneumoniae*	A, C C	(Olaitan et al., 2016) (Rolain et al., 2016)
	Malaysia	*E. coli*	A, E, C	(Yu et al., 2016)
	Pakistan	*E. coli*	C	(Mohsin et al., 2017)
	Singapore	*E. coli* *E. aerogenes* *K. pneumoniae*	C C C	(Teo et al., 2016a; Teo et al., 2016b)
	Saudi Arabia	*E. coli*	C	(Sonnevend et al., 2016)
	South Korea	*E. coli*	A	(Lim et al., 2016)
	Thailand	*E. coli*	C	(Olaitan et al., 2016)
	United Arab Emirates	*E. coli*	C	(Sonnevend et al., 2016)
	Vietnam	*E. coli* *Shigella sonnei*	A C	(Malhotra-Kumar et al., 2016b) (Pham Thanh et al., 2016)
Australia	–			
Europe	Denmark	*E. coli*	A, C	(Hasman et al., 2015)
	Estonia	*E. coli*	A	(Brauer et al., 2016)
	Germany	*E. coli*	A, C	(Falgenhauer et al., 2016)
	France	*Salmonella* spp. *E. coli* *K. pneumoniae*	A A C	(Webb et al., 2016) (Haenni et al., 2016) (Rolain et al., 2016)
	Belgium	*E. coli#*	A	(Malhotra-Kumar et al., 2016a; Xavier et al., 2016)
	Italy	*E. coli* K. pneumoniae* *Salmonella* spp.	A, C C A, C	(Cannatelli et al., 2016; Carnevali et al., 2016; Di Pilato et al., 2016; Zogg et al., 2016)
	Lithuania	*E. coli*	A	(Ruzauskas and Vaskeviciute, 2016)
	Netherlands	*E. coli*, *Salmonella* spp.	A, C A	(Arcilla et al., 2016; Kluytmans-van den Bergh et al., 2016; Veldman et al., 2016)
	Norway	*E. coli*	C	(Solheim et al., 2016)
	Poland	*E. coli*	C	(Izdebski et al., 2016)
	Portugal	*E. coli* *Salmonella* spp.	E A, C	(Campos et al., 2016; Figueiredo et al., 2016; Jones-Dias et al., 2016)
	Russia	*E. coli*	C	(Castanheira et al., 2016)
	Spain	*E. coli* *Salmonella* spp.	A, C A	(Prim et al., 2016; Quesada et al., 2016)
	Sweden	*E. coli*	C	(Vading et al., 2016)
	Switzerland	*E. coli*	E, C	(Zurfuh et al., 2016) (Nordmann et al., 2016c)
	United Kingdom	*E. coli* *S. enterica*	C A, C	(Doumith et al., 2016)
North America	Canada	*E. coli*	A, C	(Mulvey et al., 2016)
	United States of America	*E. coli*	A, C	(McGann et al., 2016; Meinersmann et al., 2016)
South America	Argentina	*E. coli*	A, C	(Liakopoulos et al., 2016; Rapoport et al., 2016)
	Brazil	*E. coli*	A	(Fernandes et al., 2016)
	Ecuador	*E. coli*	C	(Ortega-Paredes et al., 2016)
	Venezuela	*E. coli*	A, C	(Delgado-Blas et al., 2016)

A, animal sources; C, clinical sources; E, environmental sources; *mcr1.2 gene reported; #mcr-1 and mcr-2 gene reported.

#### 7.2.1 The Epidemiology of the *mcr* Gene in *Pseudomonas* spp.

**Figure 3:**
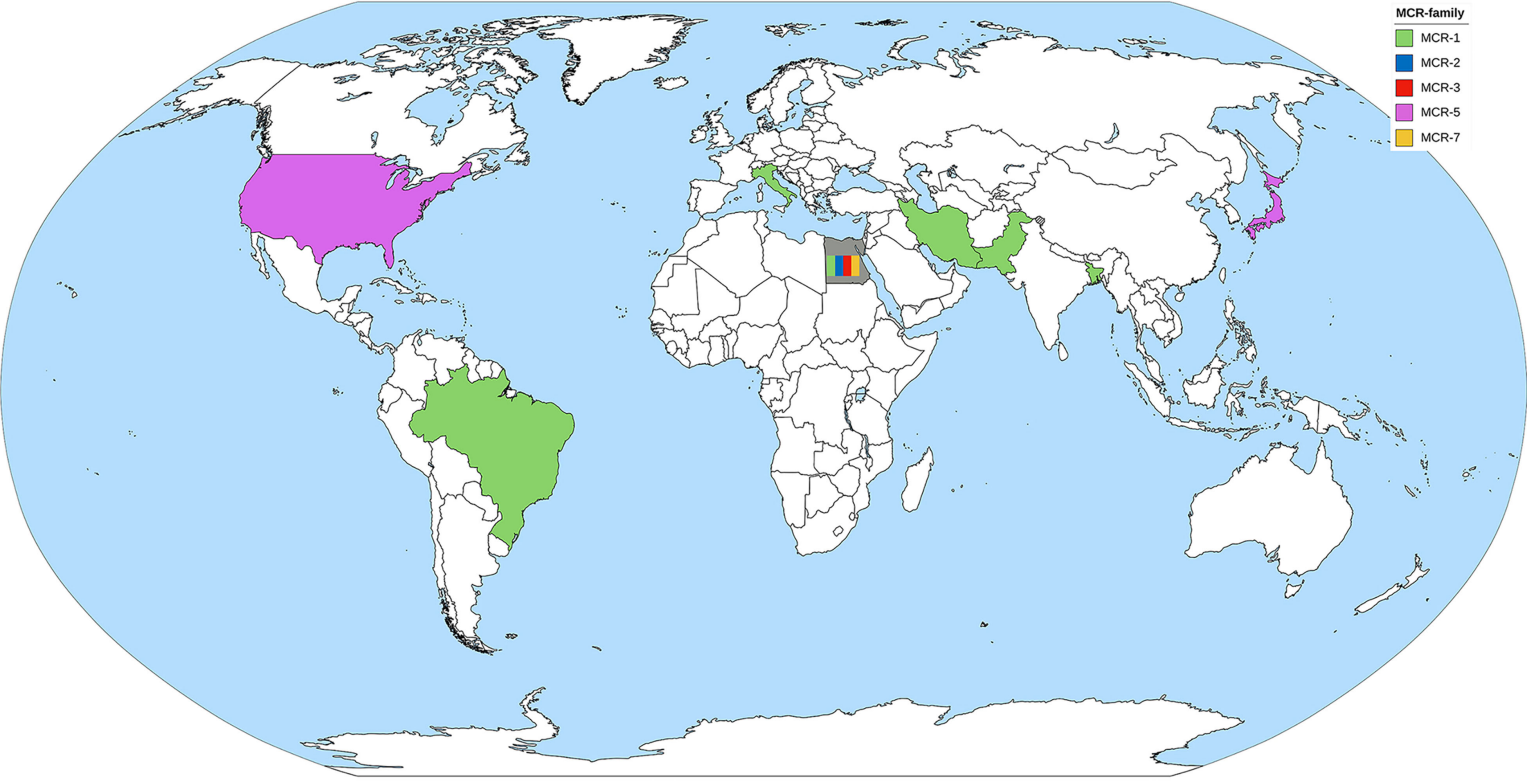
The worldwide dissemination of the *mcr* gene in *Pseudomonas* spp. Countries that reported only one type of *mcr* gene were colored to represent the *mcr* gene. The country that reported more than one type of *mcr* gene was filled with gray background containing color bands of the reported *mcr* gene.

MCR-1 is the major MCR family member found in *Pseudomonas* spp. (**Table 2**). *Pseudomonas* spp. harboring the *mcr*-gene have been reported by at least one country in all continents, except Australia (**Figure 3**). *P. aeruginosa* is a major species of *Pseudomonas* that harbors the *mcr* gene. There are also some reports of *mcr* genes in *Pseudomonas putida* (Caselli et al., 2018; Ara et al., 2021). PCR is used as the primary detection method for *mcr* genes in *Pseudomonas* spp. However, the first report of *Pseudomonas* spp. carrying the *mcr* gene by Snesrud et al. used the WGS method combining short-read and long-read sequences (Snesrud et al., 2018). Moreover, they also found that the *mcr*-5 gene was located within a Tn3-like transposon structure on the chromosome (Snesrud et al., 2018). Considering a health approach, animal and environmental sources may also be reservoirs of *mcr* genes. Ahmed et al. collected fecal samples from migratory birds in Egypt during the winter season and detected MCR-1 in *P. aeruginosa* (Ahmed et al., 2019). Some studies have detected *mcr* genes in cow’s milk, animal meat, and soil (Fujihara et al., 2015; Javed et al., 2020; Tartor et al., 2021). It is noteworthy that the oldest specimen was retrieved from the environment in 1983 but was never recognized until the WGS era (Fujihara et al., 2015). Therefore, dissemination of the *mcr* gene in the environment *via* animal hosts is another issue that needs to be considered.

**Table 2:** Summary of the *mcr* gene identified in *Pseudomonas* spp., specimen description, MCR family, and susceptibility profile.

Country	Sources of samples [details (if any)]	Detection method	Year of sample collection	Genus species	MCR family	Number of detected samples	Susceptibility profile (μg/ml) (determination method)	Reference
Bangladesh	Clinical samples (urine)	PCR	2017–2018	*P. putida*	MCR-1	3	32–128 (Agar dilution)	(Ara et al., 2021)
Brazil	Clinical sample (urine)	PCR	2015–2016	*P. aeruginosa*	MCR-1	1	≥8 (Vitek R 2 Compact)	(Nitz et al., 2021)
Brazil	Animal samples (ear swabs from cat and dog)	PCR	2018–2020	*Pseudomonas* spp.	MCR-1	11	n/a (Disk diffusion)	(Martins et al., 2022)
Egypt	Clinical samples	PCR	No data	*P. aeruginosa*	MCR-1	8	8–256 (Agar dilution)	(Abd El-Baky et al., 2020)
Egypt	Animal samples (bird feces)	PCR	2017–2018	*P. aeruginosa*	MCR-1	6	n/d	(Ahmed et al., 2019)
					MCR-2	1	n/d	
Egypt	Animal samples (milk from dairy cows)	PCR	2018–2020	*P. aeruginosa*	MCR-1	3	32–>128 (Broth microdilution)	(Tartor et al., 2021)
					MCR-2	1	128 (Broth microdilution)	
					MCR-3	3	16–64 (Broth microdilution)	
					MCR-7	1	128 (Broth microdilution)	
Egypt	Clinical sample	PCR	2019	*P. aeruginosa*	MCR-1	1	≥4 (E-test^®^)	(Shabban et al., 2020)
Iran	Clinical samples (burn and wound)	PCR	2017–2018	*P. aeruginosa*	MCR-1	3	>4 (Broth microdilution)	(Tahmasebi et al., 2020b)
Iran	Clinical samples (blood)	PCR	2018–2019	*P. aeruginosa*	MCR-1	10	≥4 (E-test^®^)	(Tahmasebi et al., 2020a)
Italy	Environment sample (hospital surfaces)	PCR	2016–2017	*P. aeruginosa*	MCR-1	1	4 (Broth microdilution)	(Caselli et al., 2018)
				*P. putida*	MCR-1	1	8 (Broth microdilution)	
Japan	Environment sample (soil)	WGS	1983	*P. aeruginosa*	MCR-5^a^	1	n/a	(Fujihara et al., 2015)
Pakistan	Clinical sample (urine)	PCR	2017–2018	*P. aeruginosa*	MCR-1	1	16 (Broth microdilution)	(Hameed et al., 2019)
Pakistan	Animal sample	PCR	No data (18 months)	*P. aeruginosa*	MCR-1	1	≥8 (SensiTest™ Colistin)	(Javed et al., 2020)
Pakistan	Clinical samples (urine, wound)	PCR	No data (6 months)	*P. aeruginosa*	MCR-1	2	≥4 (SensiTest™ Colistin)	(Ejaz et al., 2021)
United States of America	Clinical sample (wound)	WGS (short read and long read)	2012	*P. aeruginosa*	MCR-5	1	4 (Broth microdilution)	(Snesrud et al., 2018)

a Antimicrobial resistance gene database (NCBI). n/d, not determined; n/a, no data available.

Because of the low incidence of the *mcr* gene in *Pseudomonas* compared to other microorganisms in ESKAPE pathogens, this raises the question of the fitness barrier or transferability properties of the *mcr* gene among *Pseudomonas*. The transmissibility of *mcr* genes in *P. aeruginosa* was demonstrated by Tartor et al. *via* conjugation with *E. coli* J53 (Tartor et al., 2021). Four *mcr* genes, *mcr*-1, *mcr*-2, *mcr*-3, and *mcr*-7, were able to transfer into the recipient bacteria and increased the MIC of the recipient cells up to 64 μg/ml (Tartor et al., 2021). Cervoni et al. also demonstrated that MCR-1 increases colistin resistance in recipient cells. Moreover, the expression of MCR-1 in *Pseudomonas* does not affect bacterial growth or cell envelope homeostasis (Cervoni et al., 2021).

#### 7.2.2 The Epidemiology of the *mcr* Gene in *Acinetobacter* spp.

**Figure 4:**
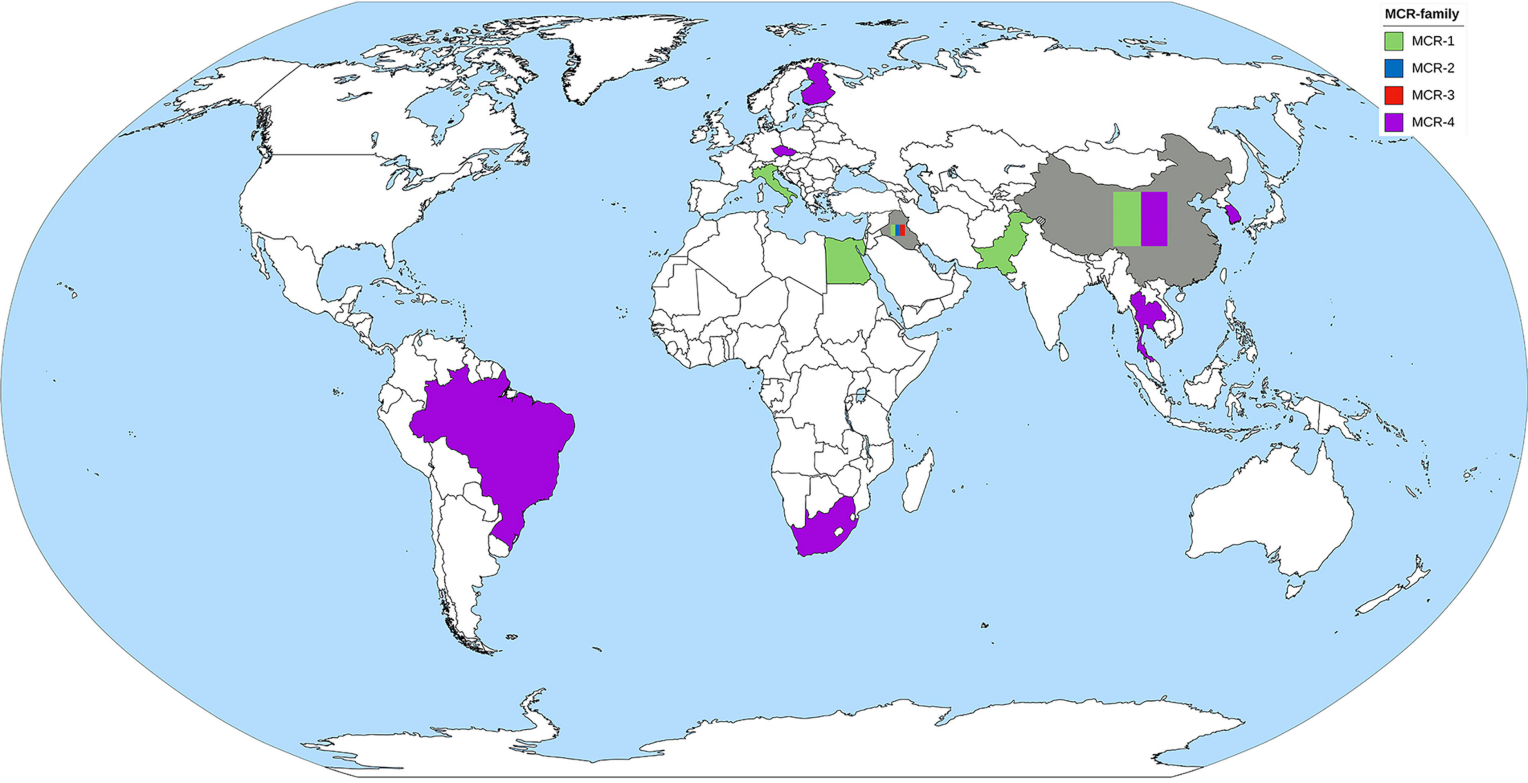
The worldwide dissemination of the *mcr* gene in *Acinetobacter* spp. Countries that reported only one type of *mcr* gene were colored to represent the *mcr* gene. Countries that reported more than one type of *mcr* gene were filled with gray background containing color bands of the reported *mcr* gene.

MCR-1 and MCR-4 are the major MCR families reported in *Acinetobacter* spp. (**Table 3**). Other *mcr* genes found in *A. baumannii* include *mcr*-2 and *mcr*-3 (Al-Kadmy et al., 2020). Reports of MCR harboring *Acinetobacter* spp. have been obtained from all continents, except North America and Australia (**Figure 4**). The oldest *Acinetobacter* specimen in which *mcr* genes were identified was from a stored clinical sample retained from a patient in Brazil in 2008, indicating that the *mcr* gene circulated for quite a period prior to its discovery by Liu et al. in 2015 (Martins-Sorenson et al., 2020). PCR has been used in *Acinetobacter* spp. for *mcr* gene detection, but short- and long-read WGS has been applied in *Acinetobacter* spp. genome studies and many mobile genetic elements involved in gene transfer have been identified (**Table 4**). While *A. baumannii* has been shown to harbor a plasmid carrying the *mcr* gene, plasmids in *A. nosocomialis* have also been reported (Cha et al., 2021; Kalova et al., 2021; Snyman et al., 2021). The *mcr*-4.3 gene in *Acinetobacter* spp. was found on a plasmid surrounding the transposon Tn3-family and/or insertion sequence. These mobile genetic elements are important for the transfer of many antibiotic resistance genes. Interestingly, some of these plasmids were unable to conjugate and/or were transferable between bacterial species.

**Table 3:** Summary of the *mcr* gene identified in *Acinetobacter* spp., specimen description, MCR family, and susceptibility profile.

Country	Sources of samples [details if any]	Detection method	Year of sample collection	Genus species	MCR family	Number of detected samples	Susceptibility profile (μg/ml) (determination method)	Reference
Brazil	Clinical sample (cerebrospinal fluid)	WGS	2008	*A. baumannii*	MCR-4.3	1	64 (Broth dilution)	(Martins-Sorenson et al., 2020)
China	Clinical sample	PCR	2018	*A. baumannii*	MCR-1.1	1	8 (Broth microdilution)	(Fan et al., 2020)
China	Animal sample (pig feces)	WGS	2018	*A. baumannii*	MCR-4.3	1	8 (Broth dilution)	(Hameed et al., 2019)
China	Animal sample (pig lung)	WGS	2018^a^	*A. pittii*	MCR-1^b^	1	n/a	(Yang and Zhang, 2018)
Czech Republic	Animals sample (imported aquaculture products)	WGS	2019	*A. baumannii*	MCR-4.3	1	>16 (Broth microdilution)	(Kalova et al., 2021)
				*A. nosocomialis*	MCR-4.3	1	>16 (Broth microdilution)	
Czech Republic	Animal sample (imported raw turkey liver)	WGS	2017	*A. baumannii*	MCR-4.3	1	16 (Broth microdilution)	(Bitar et al., 2019)
	Clinical sample (tracheal)	WGS	2017	*A. baumannii*	MCR-4.3	1	16 (Broth microdilution)	
Egypt	Clinical samples	PCR	2019	*A. baumannii*	MCR-1	2	≥4 (E-test^®^)	(Shabban et al., 2020)
Finland	Environment sample (paper pulp mill)	WGS	2020^a^	*A. baumannii*	MCR-4^b^	1	n/a	(Hamidian et al., 2020)
Iraq	Clinical samples	PCR	2014–2018	*A. baumannii*	MCR-1	22	≥4 (Broth microdilution)	(Kareem, 2020)
Iraq	Clinical and environmental samples	PCR	2016–2018	*A. baumannii*	MCR-1	89	>2 (Broth microdilution)	(Al-Kadmy et al., 2020)
					MCR-2	78		
					MCR-3	82		
Italy	Environment samples (hospital surfaces)	PCR	2016–2017	*A. lwoffii*	MCR-1	4	4-8 (Broth microdilution)	(Caselli et al., 2018)
Pakistan	Clinical sample (blood)	PCR	2017–2018	*A. baumannii*	MCR-1	1	16 (Agar dilution and broth microdilution)	(Hameed et al., 2019)
Pakistan	Clinical samples (pus, wound, tracheal)	PCR	No data (6 months)	*A. baumannii*	MCR-1	3	≥4 (SensiTest™ colistin)	(Ejaz et al., 2021)
Republic of Korea	Animal sample (imported pork)	WGS	2019	*A. nosocomialis*	MCR-4.3	1	16 (Broth microdilution)	(Cha et al., 2021)
South Africa	Clinical sample	PCR, WGS	2017	*A. nosocomialis*	MCR-4.3	1	16 (Broth microdilution and SensiTest™ Colistin)	(Snyman et al., 2021)
Thailand	Clinical sample	WGS	2010	*A. nosocomialis*	MCR-4.3^b^	1	n/a	(Kamolvit et al., 2014)

a Year of genome assembly.

b Antimicrobial resistance gene database (NCBI); n/a, no data available.

**Table 4:** Location of the *mcr* gene in *Acinetobacter* spp. and surrounding mobile genetic elements.

Bacteria strain	MCR family	Type of WGS	Gene location	Mobile genetic elements surrounding MCR gene	Year of sample collection	Reference
*A. baumannii* 597A	MCR-4.3	Short and long read	pAb-MCR4.3	-Tn3-family transposon -Insertion sequence IS*Aba19*	2008	(Martins-Sorenson et al., 2020)
*A. baumannii* LEV1449/17Ec	MCR-4.3	Short and long read	pEC_mcr4.3 (nonconjugative and nontransformable plasmid)	Insertion sequence IS*Aba19*	2017	(Bitar et al., 2019)
*A. baumannii* 39741	MCR-4.3	Short and long read	pEH _mcr4.3 (nonconjugative and nontransformable plasmid)	Insertion sequence IS*Aba19*	2017	(Bitar et al., 2019)
*A. nosocomialis CAC13*	MCR-4.3	Short and long read	Plasmid pCAC13a	-IS3 family transposase (IS*Aba19*) -Tn3 family transposase (IS*Psy42*)	2017	(Snyman et al., 2021)
*A. baumannii* AB18PR065	MCR-4.3	Short read	pAB18PR065 (nonconjugative plasmid)	Tn3 element	2018	(Hameed et al., 2019)
*A. baumannii* CT263	MCR-4.3	Short and long read	Untypeable plasmid	Tn3 family transpose IS*Psy42*	2019	(Kalova et al., 2021)
*A. nosocomialis* KUFSE-ACN036	MCR-4.3	Short read	Unidentified location due to the limitation of the short-read WGS technique	-Insertion sequence IS*Aba19* -Transposase	2019	(Kalova et al., 2021)
*A. nosocomialis* CT237	MCR-4.3	Short and long read	Untypeable plasmid	Tn3 family transpose IS*Psy42*	2019	(Cha et al., 2021)

## 8 Concluding Remarks

Colistin is recognized as a highly toxic old-generation antimicrobial agent. Owing to the shortage of antibiotic options in fighting against MDR Gram-negative bacteria, this drug was reintroduced into the clinical setting and has been recognized as one of the last-resort drugs. Non-fermentative Gram-negative bacteria such as *A. baumannii* and *P. aeruginosa* are already recognized as major threats in this century; when combined with resistance to last-resort antibiotics, the severity of the situation is critical. The resistance mechanism against colistin is considered to be chromosomally encoded and difficult to transfer. MCR was discovered in 2015, and several investigations have been subsequently published. To date, ten families of the *mcr* gene with more than 100 variants have been registered. More efforts are now being made to address this issue, and rapid detection with a high-sensitivity method is essential to track the resistance pattern. A sophisticated approach, such as WGS, is also needed to enhance the knowledge of resistance mechanisms. While waiting for the discovery of a rapid detection technique and more information, a strategy to control the resistant pathogens should be implemented along with the antibiotic stewardship program, which is recognized as a good practice in hospital settings.

## Author Contributions

PK wrote the manuscript and conceived the figures. MC and KT reviewed the manuscript draft. All authors contributed to the article and approved the submitted version.

## Funding

This work is financially supported by MU-Talents program and Specific League Funds from Mahidol University.

## Conflict of Interest

The authors declare that the research was conducted in the absence of any commercial or financial relationships that could be construed as a potential conflict of interest.

## Publisher’s Note

All claims expressed in this article are solely those of the authors and do not necessarily represent those of their affiliated organizations, or those of the publisher, the editors and the reviewers. Any product that may be evaluated in this article, or claim that may be made by its manufacturer, is not guaranteed or endorsed by the publisher.

## References

[B1] Abd El-Baky R. M. Masoud S. M. Mohamed D. S. Waly N. G. Shafik E. A. Mohareb D. A.. (2020). Prevalence and Some Possible Mechanisms of Colistin Resistance Among Multidrug-Resistant and Extensively Drug-Resistant *Pseudomonas Aeruginosa* . Infect. Drug Resist. 13, 323–332. doi: 10.2147/IDR.S238811 32099423 PMC7006860

[B2] Abdul Momin M. H. F. Bean D. C. Hendriksen R. S. Haenni M. Phee L. M. Wareham D. W. (2017). CHROMagar COL-APSE: A Selective Bacterial Culture Medium for the Isolation and Differentiation of Colistin-Resistant Gram-Negative Pathogens. J. Med. Microbiol. 66 (11), 1554–1561. doi: 10.1099/jmm.0.000602 28984232

[B3] Adams M. D. Nickel G. C. Bajaksouzian S. Lavender H. Murthy A. R. Jacobs M. R.. (2009). Resistance to Colistin in A*cinetobacter Baumannii* Associated With Mutations in the PmrAB Two-Component System. Antimicrob. Agents Chemother. 53 (9), 3628–3634. doi: 10.1128/AAC.00284-09 19528270 PMC2737849

[B4] Ahmed Z. S. Elshafiee E. A. Khalefa H. S. Kadry M. Hamza D. A. (2019). Evidence of Colistin Resistance Genes (*Mcr-1* and *Mcr-2*) in Wild Birds and its Public Health Implication in Egypt. Antimicrob. Resist. Infect. Control 8, 197. doi: 10.1186/s13756-019-0657-5 31827778 PMC6892208

[B5] Al-Kadmy I. M. S. Ibrahim S. A. Al-Saryi N. Aziz S. N. Besinis A. Hetta H. F. (2020). Prevalence of Genes Involved in Colistin Resistance in *Acinetobacter Baumannii*: First Report From Iraq. Microb. Drug Resist. 26 (6), 616–622. doi: 10.1089/mdr.2019.0243 31816255

[B6] Ara B. Urmi U. L. Haque T. A. Nahar S. Rumnaz A. Ali T.. (2021). Detection of Mobile Colistin-Resistance Gene Variants (*Mcr-1* and *Mcr-2*) in Urinary Tract Pathogens in Bangladesh: The Last Resort of Infectious Disease Management Colistin Efficacy is Under Threat. Expert Rev. Clin. Pharmacol. 14 (4), 513–522. doi: 10.1080/17512433.2021.1901577 33691556

[B7] Arcilla M. S. van Hattem J. M. Matamoros S. Melles D. C. Penders J. de Jong M. D.. (2016). Dissemination of the *Mcr-1* Colistin Resistance Gene. Lancet Infect. Dis. 16 (2), 147–149. doi: 10.1016/S1473-3099(15)00541-1 26711361

[B8] Ayoub Moubareck C. (2020). Polymyxins and Bacterial Membranes: A Review of Antibacterial Activity and Mechanisms of Resistance. Membranes (Basel) 10 (8), 181. doi: 10.3390/membranes10080181 PMC7463838 32784516

[B9] Bassetti M. Vena A. Croxatto A. Righi E. Guery B. (2018). How to Manage *Pseudomonas Aeruginosa* Infections. Drugs Context 7, 212527. doi: 10.7573/dic.212527 29872449 PMC5978525

[B10] Beceiro A. Llobet E. Aranda J. Bengoechea J. A. Doumith M. Hornsey M.. (2011). Phosphoethanolamine Modification of Lipid A in Colistin-Resistant Variants of *Acinetobacter Baumannii* Mediated by the pmrAB Two-Component Regulatory System. Antimicrob. Agents Chemother. 55 (7), 3370–3379. doi: 10.1128/AAC.00079-11 21576434 PMC3122444

[B11] Berrazeg M. Hadjadj L. Ayad A. Drissi M. Rolain J. M. (2016). First Detected Human Case in Algeria of *Mcr-1* Plasmid-Mediated Colistin Resistance in a 2011 *Escherichia Coli* Isolate. Antimicrob. Agents Chemother. 60 (11), 6996–6997. doi: 10.1128/AAC.01117-16 27572400 PMC5075129

[B12] Bialvaei A. Z. Samadi Kafil H. (2015). Colistin, Mechanisms and Prevalence of Resistance. Curr. Med. Res. Opin. 31 (4), 707–721. doi: 10.1185/03007995.2015.1018989 25697677

[B13] Bitar I. Medvecky M. Gelbicova T. Jakubu V. Hrabak J. Zemlickova H.. (2019). Complete Nucleotide Sequences of *Mcr-4.3*-Carrying Plasmids in *Acinetobacter Baumannii* Sequence Type 345 of Human and Food Origin From the Czech Republic, the First Case in Europe. Antimicrob. Agents Chemother. 63 (10), e01166-19. doi: 10.1128/AAC.01166-19 31332072 PMC6761559

[B14] Bonyadi P. Saleh N. T. Dehghani M. Yamini M. Amini K. (2022). Prevalence of Antibiotic Resistance of Pseudomonas Aeruginosa in Cystic Fibrosis Infection: A Systematic Review and Meta-Analysis. Microb. Pathog. 165, 105461. doi: 10.1016/j.micpath.2022.105461 35240288

[B15] Boucher H. W. Talbot G. H. Bradley J. S. Edwards J. E. Gilbert D. Rice L. B.. (2009). Bad Bugs, No Drugs: No ESKAPE! An Update From the Infectious Diseases Society of America. Clin. Infect. Dis. 48 (1), 1–12. doi: 10.1086/595011 19035777

[B16] Boyen F. Vangroenweghe F. Butaye P. De Graef E. Castryck F. Heylen P.. (2010). Disk Prediffusion is a Reliable Method for Testing Colistin Susceptibility in Porcine *E. Coli* Strains. Vet. Microbiol. 144 (3-4), 359–362. doi: 10.1016/j.vetmic.2010.01.010 20172663

[B17] Brauer A. Telling K. Laht M. Kalmus P. Lutsar I. Remm M.. (2016). Plasmid With Colistin Resistance Gene *Mcr-1* in Extended-Spectrum-Beta-Lactamase-Producing *Escherichia Coli* Strains Isolated From Pig Slurry in Estonia. Antimicrob. Agents Chemother. 60 (11), 6933–6936. doi: 10.1128/AAC.00443-16 27572412 PMC5075111

[B18] Cai Y. Chai D. Wang R. Liang B. Bai N. (2012). Colistin Resistance of *Acinetobacter Baumannii*: Clinical Reports, Mechanisms and Antimicrobial Strategies. J. Antimicrob. Chemother. 67 (7), 1607–1615. doi: 10.1093/jac/dks084 22441575

[B19] Campos J. Cristino L. Peixe L. Antunes P. (2016). MCR-1 in Multidrug-Resistant and Copper-Tolerant Clinically Relevant *Salmonella* 1,4,[5],12:I:- and *S.* Rissen Clones in Portuga to 2015. Euro Surveill 21 (26), pii=30270. doi: 10.2807/1560-7917.ES.2016.21.26.30270 27387036

[B20] Cannatelli A. Giani T. Antonelli A. Principe L. Luzzaro F. Rossolini G. M. (2016). First Detection of the *Mcr-1* Colistin Resistance Gene in *Escherichia Coli* in Italy. Antimicrob. Agents Chemother. 60 (5), 3257–3258. doi: 10.1128/AAC.00246-16 26976865 PMC4862502

[B21] Carnevali C. Morganti M. Scaltriti E. Bolzoni L. Pongolini S. Casadei G. (2016). Occurrence of *Mcr-1* in Colistin-Resistant *Salmonella Enterica* Isolates Recovered From Humans and Animals in Italy 2012 to 2015. Antimicrob. Agents Chemother. 60 (12), 7532–7534. doi: 10.1128/AAC.01803-16 27697756 PMC5119045

[B22] Caselli E. D’Accolti M. Soffritti I. Piffanelli M. Mazzacane S. (2018). Spread of *Mcr-1*-Driven Colistin Resistance on Hospital Surfaces, Italy. Emerg. Infect. Dis. 24 (9), 1752–1753. doi: 10.3201/eid2409.171386 30124425 PMC6106434

[B23] Castanheira M. Griffin M. A. Deshpande L. M. Mendes R. E. Jones R. N. Flamm R. K. (2016). Detection of *Mcr-1* Among *Escherichia Coli* Clinical Isolates Collected Worldwide as Part of the SENTRY Antimicrobial Surveillance Program in 2014 and 2015. Antimicrob. Agents Chemother. 60 (9), 5623–5624. doi: 10.1128/AAC.01267-16 27401568 PMC4997847

[B24] Cervoni M. Lo Sciuto A. Bianchini C. Mancone C. Imperi F. (2021). Exogenous and Endogenous Phosphoethanolamine Transferases Differently Affect Colistin Resistance and Fitness in *Pseudomonas Aeruginosa* . Front. Microbiol. 12. doi: 10.3389/fmicb.2021.778968 PMC8578941 34777328

[B25] Cha M. H. Kim S. H. Kim S. Lee W. Kwak H. S. Chi Y. M.. (2021). Antimicrobial Resistance Profile of Acinetobacter Spp. Isolates From Retail Meat Samples Under Campylobacter-Selective Conditions. J. Microbiol. Biotechnol. 31 (5), 733–739. doi: 10.4014/jmb.2102.02027 33820890 PMC9705846

[B26] Clinical and Laboratory Standards Institute (2022). Performance Standards for Antimicrobial Susceptibility Testing, 32nd Ed - CLSI supplement M100. USA: Clinical and Laboratory Standards Institute.

[B27] Dafopoulou K. Vourli S. Tsakris A. Pournaras S. (2019). An Update on Polymyxin Susceptibility Testing Methods for *Acinetobacter Baumannii* . Expert Rev. Anti Infect. Ther. 17 (9), 699–713. doi: 10.1080/14787210.2019.1667230 31509713

[B28] de Carvalho F. R. T. Telles J. P. Tuon F. F. B. Rabello Filho R. Caruso P. Correa T. D. (2022). Antimicrobial Stewardship Programs: A Review of Strategies to Avoid Polymyxins and Carbapenems Misuse in Low Middle-Income Countries. Antibiotics (Basel) 11 (3), 378. doi: 10.3390/antibiotics11030378 35326841 PMC8944697

[B29] Delgado-Blas J. F. Ovejero C. M. Abadia-Patino L. Gonzalez-Zorn B. (2016). Coexistence of *Mcr-1* and *bla*NDM*-*1 in *Escherichia Coli* From Venezuela. Antimicrob. Agents Chemother. 60 (10), 6356–6358. doi: 10.1128/AAC.01319-16 27431212 PMC5038285

[B30] Diekema D. J. Hsueh P. R. Mendes R. E. Pfaller M. A. Rolston K. V. Sader H. S.. (2019). The Microbiology of Bloodstream Infection: 20-Year Trends From the SENTRY Antimicrobial Surveillance Program. Antimicrob. Agents Chemother. 63 (7), e00355-19. doi: 10.1128/AAC.00355-19 31010862 PMC6591610

[B31] Di Pilato V. Arena F. Tascini C. Cannatelli A. Henrici De Angelis L. Fortunato S.. (2016). Mcr-1.2, A New *Mcr* Variant Carried on a Transferable Plasmid From a Colistin-Resistant KPC Carbapenemase-Producing *Klebsiella Pneumoniae* Strain of Sequence Type 512. Antimicrob. Agents Chemother. 60 (9), 5612–5615. doi: 10.1128/AAC.01075-16 27401575 PMC4997870

[B32] Doi Y. (2019). Treatment Options for Carbapenem-Resistant Gram-Negative Bacterial Infections. Clin. Infect. Dis. 69 (Suppl 7), S565–S575. doi: 10.1093/cid/ciz830 31724043 PMC6853760

[B33] Doumith M. Godbole G. Ashton P. Larkin L. Dallman T. Day M.. (2016). Detection of the Plasmid-Mediated *Mcr-1* Gene Conferring Colistin Resistance in Human and Food Isolates of *Salmonella Enterica* and *Escherichia Coli* in England and Wales. J. Antimicrob. Chemother. 71 (8), 2300–2305. doi: 10.1093/jac/dkw093 27090630

[B34] Ejaz H. Younas S. Qamar M. U. Junaid K. Abdalla A. E. Abosalif K. O. A.. (2021). Molecular Epidemiology of Extensively Drug-Resistant Mcr Encoded Colistin-Resistant Bacterial Strains Co-Expressing Multifarious Beta-Lactamases. Antibiotics (Basel) 10 (4), 467. doi: 10.3390/antibiotics10040467 33923991 PMC8073099

[B35] Elnahriry S. S. Khalifa H. O. Soliman A. M. Ahmed A. M. Hussein A. M. Shimamoto T.. (2016). Emergence of Plasmid-Mediated Colistin Resistance Gene *Mcr-1* in a Clinical *Escherichia Coli* Isolate From Egypt. Antimicrob. Agents Chemother. 60 (5), 3249–3250. doi: 10.1128/AAC.00269-16 26953204 PMC4862507

[B36] Enoch D. A. Birkett C. I. Ludlam H. A. (2007). Non-Fermentative Gram-Negative Bacteria. Int. J. Antimicrob. Agents 29 Suppl 3, S33–S41. doi: 10.1016/S0924-8579(07)72176-3 17659210

[B37] Falagas M. E. Rafailidis P. I. Matthaiou D. K. (2010). Resistance to Polymyxins: Mechanisms, Frequency and Treatment Options. Drug Resist. Update 13 (4-5), 132–138. doi: 10.1016/j.drup.2010.05.002 20843473

[B38] Falgenhauer L. Waezsada S. E. Yao Y. Imirzalioglu C. Kasbohrer A. Roesler U.. (2016). Colistin Resistance Gene *Mcr-1* in Extended-Spectrum Beta-Lactamase-Producing and Carbapenemase-Producing Gram-Negative Bacteria in Germany. Lancet Infect. Dis. 16 (3), 282–283. doi: 10.1016/S1473-3099(16)00009-8 26774242

[B39] Fan R. Li C. Duan R. Qin S. Liang J. Xiao M.. (2020). Retrospective Screening and Analysis of *Mcr-1* and *Bla* NDM in Gram-Negative Bacteria in China 2010-2019. Front. Microbiol. 11. doi: 10.3389/fmicb.2020.00121 PMC7026248 32117144

[B40] Fernandes M. R. Moura Q. Sartori L. Silva K. C. Cunha M. P. Esposito F.. (2016). Silent Dissemination of Colistin-Resistant *Escherichia Coli* in South America Could Contribute to the Global Spread of the *Mcr-1* Gene. Euro Surveill 21 (17), 30214. doi: 10.2807/1560-7917.ES.2016.21.17.30214 27168587

[B41] Fernandez L. Gooderham W. J. Bains M. McPhee J. B. Wiegand I. Hancock R. E. (2010). Adaptive Resistance to the “Last Hope” Antibiotics Polymyxin B and Colistin in *Pseudomonas Aeruginosa* is Mediated by the Novel Two-Component Regulatory System ParR-ParS. Antimicrob. Agents Chemother. 54 (8), 3372–3382. doi: 10.1128/AAC.00242-10 20547815 PMC2916309

[B42] Figueiredo R. Card R. M. Nunez J. Pomba C. Mendonca N. Anjum M. F.. (2016). Detection of an *Mcr-1*-Encoding Plasmid Mediating Colistin Resistance in *Salmonella Enterica* From Retail Meat in Portugal. J. Antimicrob. Chemother. 71 (8), 2338–2340. doi: 10.1093/jac/dkw240 27330063

[B43] Fujihara H. Yamazoe A. Hosoyama A. Suenaga H. Kimura N. Hirose J.. (2015). Draft Genome Sequence of *Pseudomonas Aeruginosa* KF702 (NBRC 110665), A Polychlorinated Biphenyl-Degrading Bacterium Isolated From Biphenyl-Contaminated Soil. Genome Announc 3 (3), e00517-15. doi: 10.1128/genomeA.00517-15 25999557 PMC4440971

[B44] Garcia-Fernandez S. Garcia-Castillo M. Ruiz-Garbajosa P. Morosini M. I. Bala Y. Zambardi G.. (2019). Performance of CHROMID(R) Colistin R Agar, A New Chromogenic Medium for Screening of Colistin-Resistant Enterobacterales. Diagn. Microbiol. Infect. Dis. 93 (1), 1–4. doi: 10.1016/j.diagmicrobio.2018.07.008 30097296

[B45] Garnacho-Montero J. Timsit J. F. (2019). Managing *Acinetobacter Baumannii* Infections. Curr. Opin. Infect. Dis. 32 (1), 69–76. doi: 10.1097/QCO.0000000000000518 30520737

[B46] Grami R. Mansour W. Mehri W. Bouallegue O. Boujaafar N. Madec J. Y.. (2016). Impact of Food Animal Trade on the Spread of *Mcr-1*-Mediated Colistin Resistance, Tunisia, July 2015. Euro Surveill 21 (8), 30144. doi: 10.2807/1560-7917.ES.2016.21.8.30144 26940999

[B47] Gutu A. D. Sgambati N. Strasbourger P. Brannon M. K. Jacobs M. A. Haugen E.. (2013). Polymyxin Resistance of *Pseudomonas Aeruginosa phoQ* Mutants is Dependent on Additional Two-Component Regulatory Systems. Antimicrob. Agents Chemother. 57 (5), 2204–2215. doi: 10.1128/AAC.02353-12 23459479 PMC3632916

[B48] Haenni M. Poirel L. Kieffer N. Chatre P. Saras E. Metayer V.. (2016). Co-Occurrence of Extended Spectrum Beta Lactamase and MCR-1 Encoding Genes on Plasmids. Lancet Infect. Dis. 16 (3), 281–282. doi: 10.1016/S1473-3099(16)00007-4 26774244

[B49] Hameed F. Khan M. A. Muhammad H. Sarwar T. Bilal H. Rehman T. U. (2019). Plasmid-Mediated *Mcr-1* Gene in *Acinetobacter Baumannii* and *Pseudomonas Aeruginosa*: First Report From Pakistan. Rev. Soc. Bras. Med. Trop. 52, e20190237. doi: 10.1590/0037-8682-0237-2019 31508785

[B50] Hamel M. Rolain J. M. Baron S. A. (2021). The History of Colistin Resistance Mechanisms in Bacteria: Progress and Challenges. Microorganisms 9 (2), 442. doi: 10.3390/microorganisms9020442 33672663 PMC7924381

[B51] Hamidian M. Maharjan R. Cain A. Faruga D. Paulsen I. (2020). *Acinetobacter Baumannii* Strain E-072658, Complete Genome.Available at: https://www.ncbi.nlm.nih.gov/nuccore/CP061705.1/ [Accessed January 31, 2022].

[B52] Hasman H. Hammerum A. M. Hansen F. Hendriksen R. S. Olesen B. Agerso Y.. (2015). Detection of *Mcr-1* Encoding Plasmid-Mediated Colistin-Resistant *Escherichia Coli* Isolates From Human Bloodstream Infection and Imported Chicken Meat, Denmark 2015. Euro Surveill 20 (49), pii=30085. doi: 10.2807/1560-7917.ES.2015.20.49.30085 26676364

[B53] Hermsen E. D. Sullivan C. J. Rotschafer J. C. (2003). Polymyxins: Pharmacology, Pharmacokinetics, Pharmacodynamics, and Clinical Applications. Infect. Dis. Clin. North Am. 17 (3), 545–562. doi: 10.1016/s0891-5520(03)00058-8 14711076

[B54] Izdebski R. Baraniak A. Bojarska K. Urbanowicz P. Fiett J. Pomorska-Wesolowska M.. (2016). Mobile MCR-1-Associated Resistance to Colistin in Poland. J. Antimicrob. Chemother. 71 (8), 2331–2333. doi: 10.1093/jac/dkw261 27330064

[B55] Javed H. Saleem S. Zafar A. Ghafoor A. Shahzad A. B. Ejaz H.. (2020). Emergence of Plasmid-Mediated *Mcr* Genes From Gram-Negative Bacteria at the Human-Animal Interface. Gut Pathog. 12 (1), 54. doi: 10.1186/s13099-020-00392-3 33292525 PMC7678191

[B56] Jean S. S. Harnod D. Hsueh P. R. (2022). Global Threat of Carbapenem-Resistant Gram-Negative Bacteria. Front. Cell Infect. Microbiol. 12. doi: 10.3389/fcimb.2022.823684 PMC8965008 35372099

[B57] Jones-Dias D. Manageiro V. Ferreira E. Barreiro P. Vieira L. Moura I. B.. (2016). Architecture of Class 1, 2, and 3 Integrons From Gram Negative Bacteria Recovered Among Fruits and Vegetables. Front. Microbiol. 7, 1400. doi: 10.3389/fmicb.2016.01400 27679611 PMC5020092

[B58] Jouy E. Haenni M. Le Devendec L. Le Roux A. Chatre P. Madec J. Y.. (2017). Improvement in Routine Detection of Colistin Resistance in *E. Coli* Isolated in Veterinary Diagnostic Laboratories. J. Microbiol. Methods 132, 125–127. doi: 10.1016/j.mimet.2016.11.017 27894831

[B59] Kalova A. Gelbicova T. Overballe-Petersen S. Litrup E. Karpiskova R. (2021). Characterisation of Colistin -Resistant *Enterobacterales* and *Acinetobacter* Strains Carrying *Mcr* Genes From Asian Aquaculture Products. Antibiotics (Basel) 10 (7), 838. doi: 10.3390/antibiotics10070838 34356760 PMC8300808

[B60] Kamolvit W. Sidjabat H. Kiratisin P. Paterson D. (2014). *Acinetobacter Nosocomialis* Strain T228, Whole Genome Shortgun Sequencing Project. Available at: https://www.ncbi.nlm.nih.gov/nuccore/JRUA00000000.1/ [Accessed January 31, 2022].

[B61] Kareem S. M. (2020). Emergence of *Mcr*- and *Fosa3*-Mediated Colistin and Fosfomycin Resistance Among Carbapenem-Resistant *Acinetobacter Baumannii* in Iraq. Meta Gene 25, 100708. doi: 10.1016/j.mgene.2020.100708

[B62] Kempf I. Fleury M. A. Drider D. Bruneau M. Sanders P. Chauvin C.. (2013). What Do We Know About Resistance to Colistin in Enterobacteriaceae in Avian and Pig Production in Europe? Int. J. Antimicrob. Agents 42 (5), 379–383. doi: 10.1016/j.ijantimicag.2013.06.012 24076115

[B63] Khalifa H. O. Ahmed A. M. Oreiby A. F. Eid A. M. Shimamoto T. Shimamoto T. (2016). Characterisation of the Plasmid-Mediated Colistin Resistance Gene *Mcr-1* in *Escherichia Coli* Isolated From Animals in Egypt. Int. J. Antimicrob. Agents 47 (5), 413–414. doi: 10.1016/j.ijantimicag.2016.02.011 27112794

[B64] Kluytmans-van den Bergh M. F. Huizinga P. Bonten M. J. Bos M. De Bruyne K. Friedrich A. W.. (2016). Presence of Mcr-1-Positive Enterobacteriaceae in Retail Chicken Meat But Not in Humans in the Netherlands Since 2009. Euro Surveill 21 (9), 30149. doi: 10.2807/1560-7917.ES.2016.21.9.30149 26967540

[B65] Kusumoto M. Ogura Y. Gotoh Y. Iwata T. Hayashi T. Akiba M. (2016). Colistin-Resistant *Mcr-1*-Positive Pathogenic *Escherichia Coli* in Swine, Japan 2007-2014. Emerg. Infect. Dis. 22 (7), 1315–1317. doi: 10.3201/eid2207.160234 27314277 PMC4918142

[B66] Letunic I. Bork P. (2021). Interactive Tree Of Life (iTOL) V5: An Online Tool for Phylogenetic Tree Display and Annotation. Nucleic Acids Res. 49 (W1), W293–W296. doi: 10.1093/nar/gkab301 33885785 PMC8265157

[B67] Liakopoulos A. Mevius D. J. Olsen B. Bonnedahl J. (2016). The Colistin Resistance *Mcr-1* Gene is Going Wild. J. Antimicrob. Chemother. 71 (8), 2335–2336. doi: 10.1093/jac/dkw262 27330067

[B68] Li X. P. Fang L. X. Song J. Q. Xia J. Huo W. Fang J. T.. (2016). Clonal Spread of Mcr-1 in PMQR-Carrying ST34 *Salmonella* Isolates From Animals in China. Sci. Rep. 6, 38511. doi: 10.1038/srep38511 27917926 PMC5137007

[B69] Lim S. K. Kang H. Y. Lee K. Moon D. C. Lee H. S. Jung S. C. (2016). First Detection of the *Mcr-1* Gene in *Escherichia Coli* Isolated From Livestock Between 2013 and 2015 in South Korea. Antimicrob. Agents Chemother. 60 (11), 6991–6993. doi: 10.1128/AAC.01472-16 27572390 PMC5075127

[B70] Lim L. M. Ly N. Anderson D. Yang J. C. Macander L. Jarkowski A. 3rd. (2010). Resurgence of Colistin: A Review of Resistance, Toxicity, Pharmacodynamics, and Dosing. Pharmacotherapy 30 (12), 1279–1291. doi: 10.1592/phco.30.12.1279 21114395 PMC4410713

[B71] Li J. Nation R. L. Turnidge J. D. Milne R. W. Coulthard K. Rayner C. R.. (2006). Colistin: The Re-Emerging Antibiotic for Multidrug-Resistant Gram-Negative Bacterial Infections. Lancet Infect. Dis. 6 (9), 589–601. doi: 10.1016/S1473-3099(06)70580-1 16931410

[B72] Liu Y. Y. Wang Y. Walsh T. R. Yi L. X. Zhang R. Spencer J.. (2016). Emergence of Plasmid-Mediated Colistin Resistance Mechanism MCR-1 in Animals and Human Beings in China: A Microbiological and Molecular Biological Study. Lancet Infect. Dis. 16 (2), 161–168. doi: 10.1016/S1473-3099(15)00424-7 26603172

[B73] Madeira F. Park Y. M. Lee J. Buso N. Gur T. Madhusoodanan N.. (2019). The EMBL-EBI Search and Sequence Analysis Tools APIs in 2019. Nucleic Acids Res. 47 (W1), W636–W641. doi: 10.1093/nar/gkz268 30976793 PMC6602479

[B74] Malhotra-Kumar S. Xavier B. B. Das A. J. Lammens C. Butaye P. Goossens H. (2016a). Colistin Resistance Gene *Mcr-1* Harboured on a Multidrug Resistant Plasmid. Lancet Infect. Dis. 16 (3), 283–284. doi: 10.1016/S1473-3099(16)00012-8 26774247

[B75] Malhotra-Kumar S. Xavier B. B. Das A. J. Lammens C. Hoang H. T. Pham N. T.. (2016b). Colistin-Resistant *Escherichia Coli* Harbouring *Mcr-1* Isolated From Food Animals in Hanoi, Vietnam. Lancet Infect. Dis. 16 (3), 286–287. doi: 10.1016/S1473-3099(16)00014-1 26774248

[B76] Mancuso G. Midiri A. Gerace E. Biondo C. (2021). Bacterial Antibiotic Resistance: The Most Critical Pathogens. Pathogens 10 (10), 1310. doi: 10.3390/pathogens10101310 34684258 PMC8541462

[B77] Martins E. Maboni G. Battisti R. da Costa L. Selva H. L. Levitzki E. D.. (2022). High Rates of Multidrug Resistance in Bacteria Associated With Small Animal Otitis: A Study of Cumulative Microbiological Culture and Antimicrobial Susceptibility. Microb. Pathog. 165, 105399. doi: 10.1016/j.micpath.2022.105399 35182615

[B78] Martins-Sorenson N. Snesrud E. Xavier D. E. Cacci L. C. Iavarone A. T. McGann P.. (2020). A Novel Plasmid-Encoded *Mcr-4.3* Gene in a Colistin-Resistant *Acinetobacter Baumannii* Clinical Strain. J. Antimicrob. Chemother. 75 (1), 60–64. doi: 10.1093/jac/dkz413 31578567 PMC6910164

[B79] McGann P. Snesrud E. Maybank R. Corey B. Ong A. C. Clifford R.. (2016). *Escherichia Coli* Harboring *Mcr-1* and *bla*CTX*-*M on a Novel IncF Plasmid: First Report of *Mcr-1* in the United States. Antimicrob. Agents Chemother. 60 (7), 4420–4421. doi: 10.1128/AAC.01103-16 27230792 PMC4914657

[B80] McPhee J. B. Lewenza S. Hancock R. E. (2003). Cationic Antimicrobial Peptides Activate a Two-Component Regulatory System, PmrA-PmrB, That Regulates Resistance to Polymyxin B and Cationic Antimicrobial Peptides in *Pseudomonas Aeruginosa* . Mol. Microbiol. 50 (1), 205–217. doi: 10.1046/j.1365-2958.2003.03673.x 14507375

[B81] Meinersmann R. J. Ladely S. R. Plumblee J. R. Hall M. C. Simpson S. A. Ballard L. L.. (2016). Colistin Resistance *Mcr-1*-Gene-Bearing *Escherichia Coli* Strain From the United States. Genome Announc 4 (5), e00 898-16. doi: 10.1128/genomeA.00898-16 PMC5009973 27587816

[B82] Micek S. T. Wunderink R. G. Kollef M. H. Chen C. Rello J. Chastre J.. (2015). An International Multicenter Retrospective Study of *Pseudomonas Aeruginosa* Nosocomial Pneumonia: Impact of Multidrug Resistance. Crit. Care 19, 219. doi: 10.1186/s13054-015-0926-5 25944081 PMC4446947

[B83] Moffatt J. H. Harper M. Adler B. Nation R. L. Li J. Boyce J. D. (2011). Insertion Sequence ISAba11 is Involved in Colistin Resistance and Loss of Lipopolysaccharide in *Acinetobacter Baumannii* . Antimicrob. Agents Chemother. 55 (6), 3022–3024. doi: 10.1128/AAC.01732-10 21402838 PMC3101452

[B84] Moffatt J. H. Harper M. Harrison P. Hale J. D. Vinogradov E. Seemann T.. (2010). Colistin Resistance in *Acinetobacter Baumannii* is Mediated by Complete Loss of Lipopolysaccharide Production. Antimicrob. Agents Chemother. 54 (12), 4971–4977. doi: 10.1128/AAC.00834-10 20855724 PMC2981238

[B85] Mohsin M. Raza S. Roschanski N. Guenther S. Ali A. Schierack P. (2017). Description of the First *Escherichia Coli* Clinical Isolate Harboring the Colistin Resistance Gene *Mcr-1* From the Indian Subcontinent. Antimicrob. Agents Chemother. 61 (1), e01945-16. doi: 10.1128/AAC.01945-16 27795381 PMC5192098

[B86] Mulvey M. R. Mataseje L. F. Robertson J. Nash J. H. Boerlin P. Toye B.. (2016). Dissemination of the *Mcr-1* Colistin Resistance Gene. Lancet Infect. Dis. 16 (3), 289–290. doi: 10.1016/S1473-3099(16)00067-0 26973304

[B87] National Antimicrobial Resistant Surveillance Center, T (2020). Antibiogram 2019 (Jan - Dec). Available at: http://narst.dmsc.moph.go.th/antibiograms.html [Accessed January 20, 2022].

[B88] National Library of Medicine (2022). NCBI National Database of Antibiotic Resistant Organisms (NDARO). Available at: https://www.ncbi.nlm.nih.gov/pathogens/antimicrobial-resistance/ [Accessed January 31, 2022].

[B89] Nation R. L. Li J. (2009). Colistin in the 21st Century. Curr. Opin. Infect. Dis. 22 (6), 535–543. doi: 10.1097/QCO.0b013e328332e672 19797945 PMC2869076

[B90] Needham B. D. Trent M. S. (2013). Fortifying the Barrier: The Impact of Lipid A Remodelling on Bacterial Pathogenesis. Nat. Rev. Microbiol. 11 (7), 467–481. doi: 10.1038/nrmicro3047 23748343 PMC6913092

[B91] Nitz F. de Melo B. O. da Silva L. C. N. de Souza Monteiro A. Marques S. G. Monteiro-Neto V.. (2021). Molecular Detection of Drug-Resistance Genes of *bla*OXA*-*23-*bla*OXA*-*51 and *Mcr-1* in Clinical Isolates of *Pseudomonas Aeruginosa* . Microorganisms 9 (4), 786. doi: 10.3390/microorganisms9040786 33918745 PMC8069495

[B92] Nordmann P. Jayol A. Poirel L. (2016a). Rapid Detection of Polymyxin Resistance in Enterobacteriaceae. Emerg. Infect. Dis. 22 (6), 1038–1043. doi: 10.3201/eid2206.151840 27191712 PMC4880072

[B93] Nordmann P. Jayol A. Poirel L. (2016b). A Universal Culture Medium for Screening Polymyxin-Resistant Gram-Negative Isolates. J. Clin. Microbiol. 54 (5), 1395–1399. doi: 10.1128/JCM.00446-16 26984971 PMC4844728

[B94] Nordmann P. Lienhard R. Kieffer N. Clerc O. Poirel L. (2016c). Plasmid-Mediated Colistin-Resistant *Escherichia Coli* in Bacteremia in Switzerland. Clin. Infect. Dis. 62 (10), 1322–1323. doi: 10.1093/cid/ciw124 26936673

[B95] Olaitan A. O. Chabou S. Okdah L. Morand S. Rolain J. M. (2016). Dissemination of the *Mcr-1* Colistin Resistance Gene. Lancet Infect. Dis. 16 (2), 147. doi: 10.1016/S1473-3099(15)00540-X 26711360

[B96] Olaitan A. O. Morand S. Rolain J. M. (2014). Mechanisms of Polymyxin Resistance: Acquired and Intrinsic Resistance in Bacteria. Front. Microbiol. 5. doi: 10.3389/fmicb.2014.00643 PMC4244539 25505462

[B97] Ortega-Paredes D. Barba P. Zurita J. (2016). Colistin-Resistant *Escherichia Coli* Clinical Isolate Harbouring the *Mcr-1* Gene in Ecuador. Epidemiol. Infect. 144 (14), 2967–2970. doi: 10.1017/S0950268816001369 27586373 PMC9150395

[B98] Partridge S. R. Di Pilato V. Doi Y. Feldgarden M. Haft D. H. Klimke W.. (2018). Proposal for Assignment of Allele Numbers for Mobile Colistin Resistance (*Mcr*) Genes. J. Antimicrob. Chemother. 73 (10), 2625–2630. doi: 10.1093/jac/dky262 30053115 PMC6148208

[B99] Perreten V. Strauss C. Collaud A. Gerber D. (2016). Colistin Resistance Gene *Mcr-1* in Avian-Pathogenic *Escherichia Coli* in South Africa. Antimicrob. Agents Chemother. 60 (7), 4414–4415. doi: 10.1128/AAC.00548-16 27161625 PMC4914693

[B100] Pham Thanh D. Thanh Tuyen H. Nguyen Thi Nguyen T. Chung The H. Wick R. R. Thwaites G. E.. (2016). Inducible Colistin Resistance *via* a Disrupted Plasmid-Borne Mcr-1 Gene in a 2008 Vietnamese *Shigella Sonnei* Isolate. J. Antimicrob. Chemother. 71 (8), 2314–2317. doi: 10.1093/jac/dkw173 27246235 PMC4954929

[B101] Poirel L. Kieffer N. Brink A. Coetze J. Jayol A. Nordmann P. (2016). Genetic Features of MCR-1-Producing Colistin-Resistant *Escherichia Coli* Isolates in South Africa. Antimicrob. Agents Chemother. 60 (7), 4394–4397. doi: 10.1128/AAC.00444-16 27161623 PMC4914673

[B102] Prim N. Rivera A. Rodriguez-Navarro J. Espanol M. Turbau M. Coll P.. (2016). Detection of *Mcr-1* Colistin Resistance Gene in Polyclonal *Escherichia Coli* Isolates in Barcelona, Spain 2012 to 2015. Euro Surveill 21 (13), pii=30183. doi: 10.2807/1560-7917.ES.2016.21.13.30183 27055477

[B103] Quesada A. Ugarte-Ruiz M. Iglesias M. R. Porrero M. C. Martinez R. Florez-Cuadrado D.. (2016). Detection of Plasmid Mediated Colistin Resistance (MCR-1) in *Escherichia Coli* and *Salmonella Enterica* Isolated From Poultry and Swine in Spain. Res. Vet. Sci. 105, 134–135. doi: 10.1016/j.rvsc.2016.02.003 27033921

[B104] Ramirez M. S. Bonomo R. A. Tolmasky M. E. (2020). Carbapenemases: Transforming *Acinetobacter Baumannii* Into a Yet More Dangerous Menace. Biomolecules 10 (5), 720. doi: 10.3390/biom10050720 PMC7277208 32384624

[B105] Rapoport M. Faccone D. Pasteran F. Ceriana P. Albornoz E. Petroni A.. (2016). First Description of *Mcr-1*-Mediated Colistin Resistance in Human Infections Caused by *Escherichia Coli* in Latin America. Antimicrob. Agents Chemother. 60 (7), 4412–4413. doi: 10.1128/AAC.00573-16 27090181 PMC4914662

[B106] Rhouma M. Beaudry F. Letellier A. (2016a). Resistance to Colistin: What is the Fate for This Antibiotic in Pig Production? Int. J. Antimicrob. Agents 48 (2), 119–126. doi: 10.1016/j.ijantimicag.2016.04.008 27234675

[B107] Rhouma M. Beaudry F. Theriault W. Letellier A. (2016b). Colistin in Pig Production: Chemistry, Mechanism of Antibacterial Action, Microbial Resistance Emergence, and One Health Perspectives. Front. Microbiol. 7. doi: 10.3389/fmicb.2016.01789 PMC5104958 27891118

[B108] Rolain J. M. Kempf M. Leangapichart T. Chabou S. Olaitan A. O. Le Page S.. (2016). Plasmid-Mediated *Mcr-1* Gene in Colistin-Resistant Clinical Isolates of *Klebsiella Pneumoniae* in France and Laos. Antimicrob. Agents Chemother. 60 (11), 6994–6995. doi: 10.1128/AAC.00960-16 27572402 PMC5075128

[B109] Ruzauskas M. Vaskeviciute L. (2016). Detection of the *Mcr-1* Gene in *Escherichia Coli* Prevalent in the Migratory Bird Species Larus Argentatus. J. Antimicrob. Chemother. 71 (8), 2333–2334. doi: 10.1093/jac/dkw245 27330066

[B110] Shabban M. Fahim N. A. E. Montasser K. Abo El Magd N. M. (2020). Resistance to Colistin Mediated by *Mcr-1* Among Multidrug Resistant Gram Negative Pathogens at a Tertiary Care Hospital, Egypt. J. Pure Appl. Microbiol. 14 (2), 1125–1132. doi: 10.22207/JPAM.14.2.07

[B111] Snesrud E. Maybank R. Kwak Y. I. Jones A. R. Hinkle M. K. McGann P. (2018). Chromosomally Encoded *Mcr-5* in Colistin-Nonsusceptible *Pseudomonas Aeruginosa* . Antimicrob. Agents Chemother. 62 (8), e00679-18. doi: 10.1128/AAC.00679-18 29844041 PMC6105811

[B112] Snyman Y. Reuter S. Whitelaw A. C. Stein L. Maloba M. R. B. Newton-Foot M. (2021). Characterisation of *Mcr-4.3* in a Colistin-Resistant *Acinetobacter Nosocomialis* Clinical Isolate From Cape Town, South Africa. J. Glob Antimicrob. Resist. 25, 102–106. doi: 10.1016/j.jgar.2021.03.002 33757821

[B113] Solheim M. Bohlin J. Ulstad C. R. Schau Slettemeas J. Naseer U. Dahle U. R.. (2016). Plasmid-Mediated Colistin-Resistant Escherichia Coli Detected From 2014 in Norway. Int. J. Antimicrob. Agents 48 (2), 227–228. doi: 10.1016/j.ijantimicag.2016.06.001 27388575

[B114] Sonnevend A. Ghazawi A. Alqahtani M. Shibl A. Jamal W. Hashmey R.. (2016). Plasmid-Mediated Colistin Resistance in *Escherichia Coli* From the Arabian Peninsula. Int. J. Infect. Dis. 50, 85–90. doi: 10.1016/j.ijid.2016.07.007 27566913

[B115] Stoesser N. Mathers A. J. Moore C. E. Day N. P. Crook D. W. (2016). Colistin Resistance Gene *Mcr-1* and Phnshp45 Plasmid in Human Isolates of *Escherichia Coli* and *Klebsiella Pneumoniae* . Lancet Infect. Dis. 16 (3), 285–286. doi: 10.1016/S1473-3099(16)00010-4 26774239

[B116] Suzuki S. Ohnishi M. Kawanishi M. Akiba M. Kuroda M. (2016). Investigation of a Plasmid Genome Database for Colistin-Resistance Gene *Mcr-1* . Lancet Infect. Dis. 16 (3), 284–285. doi: 10.1016/S1473-3099(16)00008-6 26774245

[B117] Sy C. L. Chen P. Y. Cheng C. W. Huang L. J. Wang C. H. Chang T. H.. (2022). Recommendations and Guidelines for the Treatment of Infections Due to Multidrug Resistant Organisms. J. Microbiol. Immunol. Infect. doi: 10.1016/j.jmii.2022.02.001. (Accessed April 20, 2022). 35370082

[B118] Tahmasebi H. Dehbashi S. Arabestani M. R. (2020a). Co-Harboring of *Mcr-1* and Beta-Lactamase Genes in *Pseudomonas Aeruginosa* by High-Resolution Melting Curve Analysis (HRMA): Molecular Typing of Superbug Strains in Bloodstream Infections (BSI). Infect. Genet. Evol. 85, 104518. doi: 10.1016/j.meegid.2020.104518 32891877

[B119] Tahmasebi H. Dehbashi S. Arabestani M. R. (2020b). Prevalence and Molecular Typing of Colistin-Resistant *Pseudomonas Aeruginosa* (CRPA) Among Beta-Lactamase-Producing Isolates: A Study Based on High-Resolution Melting Curve Analysis Method. Infect. Drug Resist. 13, 2943–2955. doi: 10.2147/IDR.S264796 32922046 PMC7457805

[B120] Tartor Y. H. Gharieb R. M. A. Abd El-Aziz N. K. El Damaty H. M. Enany S. Khalifa E.. (2021). Virulence Determinants and Plasmid-Mediated Colistin Resistance *Mcr* Genes in Gram-Negative Bacteria Isolated From Bovine Milk. Front. Cell Infect. Microbiol. 11. doi: 10.3389/fcimb.2021.761417 PMC8650641 34888259

[B121] Teo J. W. Chew K. L. Lin R. T. (2016b). Transmissible Colistin Resistance Encoded by *Mcr-1* Detected in Clinical Enterobacteriaceae Isolates in Singapore. Emerg. Microbes Infect. 5 (8), e87. doi: 10.1038/emi.2016.85 27530747 PMC5034101

[B122] Teo J. Q. Ong R. T. Xia E. Koh T. H. Khor C. C. Lee S. J.. (2016a). *Mcr-1* in Multidrug-Resistant *bla*KPC*-*2-Producing Clinical *Enterobacteriaceae* Isolates in Singapore. Antimicrob. Agents Chemother. 60 (10), 6435–6437. doi: 10.1128/AAC.00804-16 27503652 PMC5038244

[B123] The European Committee on Antimicrobial Susceptibility Testing (2022). Clinical Breakpoints - Breakpoints and Guidance. Available at: https://www.eucast.org/clinical_breakpoints/ [Accessed January 20, 2022].

[B124] Tsuji B. T. Pogue J. M. Zavascki A. P. Paul M. Daikos G. L. Forrest A.. (2019). International Consensus Guidelines for the Optimal Use of the Polymyxins: Endorsed by the American College of Clinical Pharmacy (ACCP), European Society of Clinical Microbiology and Infectious Diseases (ESCMID), Infectious Diseases Society of America (IDSA), International Society for Anti-Infective Pharmacology (ISAP), Society of Critical Care Medicine (SCCM), and Society of Infectious Diseases Pharmacists (SIDP). Pharmacotherapy 39 (1), 10–39. doi: 10.1002/phar.2209 30710469 PMC7437259

[B125] Vading M. Kabir M. H. Kalin M. Iversen A. Wiklund S. Naucler P.. (2016). Frequent Acquisition of Low-Virulence Strains of ESBL-Producing *Escherichia Coli* in Travellers. J. Antimicrob. Chemother. 71 (12), 3548–3555. doi: 10.1093/jac/dkw335 27566312

[B126] Veldman K. van Essen-Zandbergen A. Rapallini M. Wit B. Heymans R. van Pelt W.. (2016). Location of Colistin Resistance Gene *Mcr-1* in Enterobacteriaceae From Livestock and Meat. J. Antimicrob. Chemother. 71 (8), 2340–2342. doi: 10.1093/jac/dkw181 27246233

[B127] Webb H. E. Granier S. A. Marault M. Millemann Y. den Bakker H. C. Nightingale K. K.. (2016). Dissemination of the *Mcr-1* Colistin Resistance Gene. Lancet Infect. Dis. 16 (2), 144–145. doi: 10.1016/S1473-3099(15)00538-1 26711363

[B128] WHO Regional Office for Europe/European Centre for Disease Prevention and Control (2022). Antimicrobial Resistance Surveillance in Europe 2022 - 2020 data. Copenhagen: WHO Regional Office for Europe.

[B129] World Health Organization (2017) WHO Publishes List of Bacteria for Which New Antibiotics are Urgently Needed. Available at: https://www.who.int/news/item/27-02-2017-who-publishes-list-of-bacteria-for-which-new-antibiotics-are-urgently-needed (Accessed December 19, 2021).

[B130] World Health Organization (2020). GLASS Whole-Genome Sequencing for Surveillance of Antimicrobial Resistance. Available at: https://www.who.int/publications/i/item/9789240011007 [Accessed January 20, 2022].

[B131] World Health Organization (2021). GLASS: The Detection and Reporting of Colistin Resistance. Available at: https://www.who.int/publications/i/item/glass-the-detection-and-reporting-of-colistin-resistance-2nd-ed [Accessed January 20, 2022].

[B132] Xavier B. B. Lammens C. Ruhal R. Kumar-Singh S. Butaye P. Goossens H.. (2016). Identification of a Novel Plasmid-Mediated Colistin-Resistance Gene, *Mcr-2*, in *Escherichia Coli*, Belgium, June 2016. Euro Surveill 21 (27), 30280. doi: 10.2807/1560-7917.ES.2016.21.27.30280 27416987

[B133] Yang Y. Zhang A. (2018). Acinetobacter Pittii Strain SCsI25, Whole Genome Shortgun Sequencing Project. https://www.ncbi.nlm.nih.gov/nuccore/VDIH00000000.1/ [Accessed January 31, 2022].

[B134] Yu C. Y. Ang G. Y. Chin P. S. Ngeow Y. F. Yin W. F. Chan K. G. (2016). Emergence of *Mcr-1*-Mediated Colistin Resistance in *Escherichia Coli* in Malaysia. Int. J. Antimicrob. Agents 47 (6), 504–505. doi: 10.1016/j.ijantimicag.2016.04.004 27208898

[B135] Zeng K. J. Doi Y. Patil S. Huang X. Tian G. B. (2016). Emergence of the Plasmid-Mediated *Mcr-1* Gene in Colistin-Resistant *Enterobacter Aerogenes* and *Enterobacter Cloacae* . Antimicrob. Agents Chemother. 60 (6), 3862–3863. doi: 10.1128/AAC.00345-16 26976876 PMC4879368

[B136] Zhanel G. G. Adam H. J. Baxter M. R. Fuller J. Nichol K. A. Denisuik A. J.. (2019). 42936 Pathogens From Canadian Hospitals: 10 Years of Results,(2017-16) From the CANWARD Surveillance Study. J. Antimicrob. Chemother. 74 (Suppl 4), iv5–iv21. doi: 10.1093/jac/dkz283 31505641

[B137] Zhang H. Srinivas S. Xu Y. Wei W. Feng Y. (2019). Genetic and Biochemical Mechanisms for Bacterial Lipid A Modifiers Associated With Polymyxin Resistance. Trends Biochem. Sci. 44 (11), 973–988. doi: 10.1016/j.tibs.2019.06.002 31279652

[B138] Zhao F. Zong Z. (2016). *Kluyvera Ascorbata* Strain From Hospital Sewage Carrying the *Mcr-1* Colistin Resistance Gene. Antimicrob. Agents Chemother. 60 (12), 7498–7501. doi: 10.1128/AAC.01165-16 27671069 PMC5119035

[B139] Zogg A. L. Zurfluh K. Nuesch-Inderbinen M. Stephan R. (2016). Characteristics of ESBL-Producing Enterobacteriaceae and Methicillinresistant *Staphylococcus Aureus* (MRSA) Isolated From Swiss and Imported Raw Poultry Meat Collected at Retail Level. Schweiz Arch. Tierheilkd 158 (6), 451–456. doi: 10.17236/sat00071 27504840

[B140] Zurfuh K. Poirel L. Nordmann P. Nuesch-Inderbinen M. Hachler H. Stephan R. (2016). Occurrence of the Plasmid-Borne Mcr-1 Colistin Resistance Gene in Extended-Spectrum-Beta-Lactamase-Producing Enterobacteriaceae in River Water and Imported Vegetable Samples in Switzerland. Antimicrob. Agents Chemother. 60 (4), 2594–2595. doi: 10.1128/AAC.00066-16 26883696 PMC4808203

